# Lactoferrin and Nanotechnology: New Perspectives and Therapeutic Applications for This Multifunctional Protein

**DOI:** 10.3390/ijms27209226

**Published:** 2026-10-17

**Authors:** Fabiana Superti, Alessia Mariano, Anna Scotto d’Abusco

**Affiliations:** 1 Association for Research on Integrative Oncology Therapies, (ARTOI) Foundation, Via Ludovico Micara, 73, 00165 Rome, RM, Italy; 2 Department of Biochemical Sciences, Sapienza University of Rome, 00185 Rome, RM, Italy; alessia.mariano@uniroma1.it (A.M.); anna.scottodabusco@uniroma1.it (A.S.d.); 3 BeSSA Department of Wellbeing, Health and Environmental Sustainability, Sapienza University of Rome, 02100 Rieti, RI, Italy

**Keywords:** lactoferrin, nanoparticles, drug delivery, biocompatibility, noncommunicable diseases, communicable diseases, nanomedicine

## Abstract

Lactoferrin, a multifunctional glycoprotein belonging to the transferrin family, is proving to be an extremely promising drug delivery vehicle, with numerous applications in the field of nanotechnology. This protein is biocompatible and well-tolerated and, regarding cell targeting, it is capable of binding to low-density lipoprotein receptor-related protein 1, a large multifunctional transmembrane receptor that mediates endocytosis, cell signaling, and protein clearance. Interestingly, lipoprotein receptor-related protein 1 is overexpressed in various cell types, particularly on tumor cells and blood–brain barrier endothelial cells. The purpose of this review is to summarize the most important beneficial activities demonstrated by lactoferrin nanoparticles obtained through various methods, aiming to identify potential prospects for their future applications in nanomedicine. Due to its remarkable stability and safety, interaction with specific cellular receptors, and ability to cross various key biological barriers in the human body, including the blood–brain barrier, lactoferrin has been successfully used to deliver therapeutic agents directly to their targets. This reduces systemic side effects while improving bioavailability and therapeutic outcomes. The main specific characteristics of lactoferrin nanoparticles include controlled release of the active ingredient at acidic pH, the ability to deliver hydrophobic drugs through various routes, and the greater efficacy and lower toxicity of the encapsulated drug compared to the free drug. This review highlights scientific evidence from in vitro and in vivo studies regarding the benefits of lactoferrin-based nanoformulations to enhance the therapeutic efficacy of molecules and drugs used to treat communicable and noncommunicable diseases. However, given that their practical application has remained rather limited to date, it is anticipated that lactoferrin nanoparticles will increasingly attract the attention of researchers across various fields, such as biology, medicine, and nanotechnology, with the aim of enabling their clinical application.

## 1. Introduction

**Figure 1:**
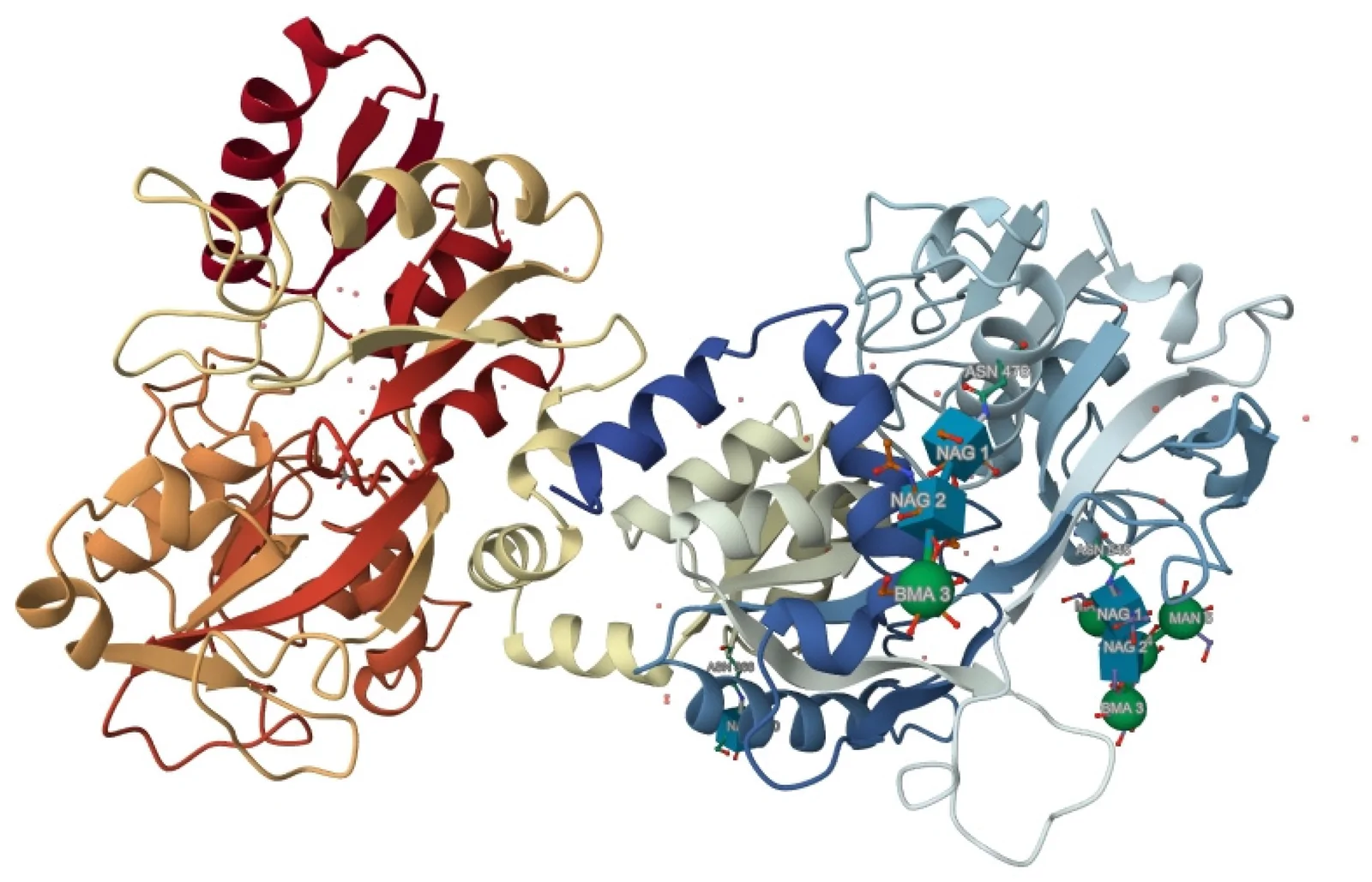
Three-dimensional crystal structure and glycosylation profiling of bovine lactoferrin. The ribbon diagram illustrates the bi-lobular architecture of the protein, divided into the N-lobe (left, represented in red/orange shades) and the C-lobe (right, represented in blue/light-blue shades), connected by the central α-helical spacer (cream). The right section highlights the post-translational modification network, showcasing the explicit N-glycosylation sites attached to asparagine residues (e.g., ASN 476). Major carbohydrate moieties, including N-acetylglucosamine (NAG 1, NAG 2, cyan squares), β-d-mannose (BMA 3, green spheres), and α-d-mannose (MAN, green hexagon), are structurally mapped to demonstrate the glycan branching that acts as a ligand for lectin receptors. The structural model was retrieved from the Protein Data Bank (PDB ID: 1BLF).

Lactoferrin (Lf) is a multifunctional glycoprotein of approximately 80 kDa belonging to the transferrin family. It is present in milk and other exocrine secretions such as tears, saliva, nasal and bronchial secretions, vaginal mucus, seminal fluid, bile, gastrointestinal fluids, and urine [1]. It is also present in plasma, where it is derived predominantly from neutrophils [2]. Lf has numerous therapeutic applications in both infectious and noncommunicable diseases, primarily due to its unique structural characteristics, specific interactions with target cells, biocompatibility, and bioavailability. Regarding its structural characteristics, Lf is a single-chain glycoprotein consisting of two homologous lobes (the N lobe and the C lobe), joined by a hinge region. Each lobe can bind a ferric ion (Fe^3+^) in coordination with a bicarbonate anion (HCO_3_^−^), giving Lf its ability to chelate iron. This capability is essential for Lf to exert its antimicrobial activity by sequestering iron from pathogenic microorganisms. The protein surface is also rich in glycosylation sites, which facilitate the binding to specific cellular receptors. These receptors include low-density lipoprotein receptor-related protein 1 (LRP1), intelectin-1 (ITLN1), and Lf-specific receptors such as Lf Receptor (LfR). Specifically, Lf N- and C-lobes expose specific peptide sequences required for high-affinity binding to low-density LRP1 and LfR, whereas N-glycosylation acts as the primary stereospecific ligand for ITLN1 receptor [3,4] (Figure 1).

The receptor-mediated interactions are particularly important as they allow Lf to cross biological barriers. Lf is, in fact, capable of crossing both the intestinal epithelium and the blood–brain barrier (IEB and BBB). This characteristic plays a key role in drug delivery, a process in which targeted release and enhanced permeability are crucial factors [5]. Other fundamental characteristics of Lf, which is intrinsically non-toxic, are its biocompatibility and bioavailability. This glycoprotein is also able to protect cells against oxidative stress and possesses anti-inflammatory and immunomodulatory activities, preventing the formation of free radicals and regulating the production of cytokines and the activity of immune cells [6].

Due to these features, Lf has found numerous applications in the field of nanotechnology; among the most significant are the direct incorporation of drugs into Lf, primarily of bovine origin, nanoparticles (NPs) and the conjugation of Lf with preformed NPs. The ultimate goal is to coat NPs with Lf and direct them to the site of action, thereby improving the bioavailability of the transported drugs.

These Lf-based nanocarriers can function as active therapeutic agents and, crucially, as vehicles for targeted drug delivery, finding diverse medical applications in the fields of both communicable and noncommunicable diseases. For example, in infectious diseases, Lf NPs are used for their intrinsic ability to sequester iron from pathogenic bacteria, enhancing the effectiveness of loaded antibiotics. Moreover, due to their ability to cross the BBB via receptor-mediated endocytosis, Lf NPs are currently being studied for drug delivery to the brain for the treatment of neurodegenerative disorders, such as Alzheimer’s and Parkinson’s diseases, or brain tumors such as gliomas and glioblastoma. To date, various types of Lf NPs have been developed, resulting in improved drug stability, increased bioavailability, and effective tissue-specific drug targeting [7,8].

This review is grounded on the hypothesis that the unique structural and functional properties of bovine lactoferrin make Lf-based nanocarriers a game-changing strategy to enhance targeted therapy across a broad range of human pathologies. To evaluate this rationale and provide a comprehensive overview of recent advancements, we summarize the various techniques for preparing Lf-based nanocarriers, along with the most important works and their different types of applications. The advantages and disadvantages of these nano-preparations will also be discussed to provide insights that may pave the way for the practical use of these technologies in the fight against communicable and noncommunicable diseases.

## 2. Preparation Techniques of Lactoferrin Nanocarriers

The unique properties of bovine lactoferrin (Lf) have been exploited in numerous medical applications, including its use as self-assembling, pH-sensitive NPs to specifically deliver drugs to the target site of a disease. Lf NPs are produced using methods such as sol-oil or water-in-oil emulsion methods, thermal denaturation (heat treatment) procedure, desolvation process, albumin-bound NPs (Nab) technology, combinations of oppositely charged molecules to form electrostatic nanocomplexes, Lf self-assembly process, Lf-drug nano-conjugated method, Lf shell-oil core technique, and green synthesis using Lf as a “corona” to stabilize inorganic NPs.

### 2.1. Sol-Oil or Water-in-Oil Emulsion Method

**Figure 2:**
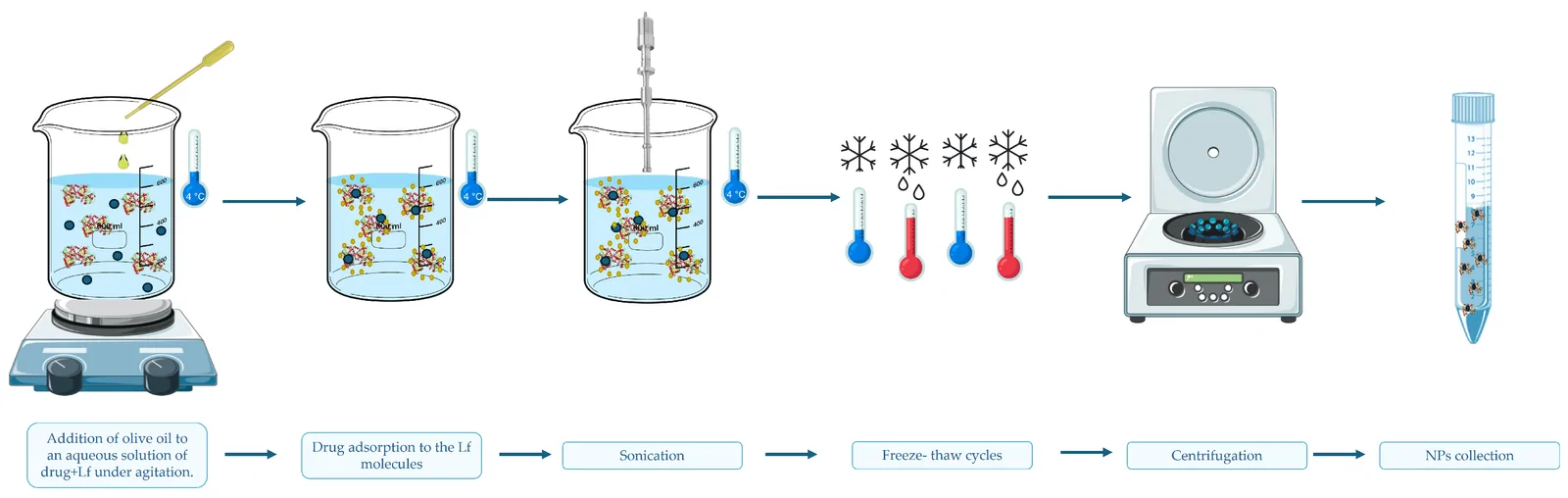
Preparation of lactoferrin nanoparticles by sol-oil or water-in-oil emulsion method.

Sol-oil (or water-in-oil) emulsions are widely used to encapsulate bioactive compounds in aqueous solutions and represent one of the most common methods utilized for producing Lf NPs. The NP preparation procedure involves the slow addition of olive oil to an aqueous solution of drug-Lf mixture at 4 °C under continuous stirring. This allows the drug to adsorb onto the Lf molecules, forming emulsified aggregates. After sonication, the aggregates are frozen in liquid nitrogen and then thawed. The resulting NPs are collected by centrifugation, washed, and dispersed in an appropriate medium (Figure 2).

As shown in Table 1 and Table 2, several anticancer and antiviral (primarily antiretroviral) drugs have been successfully loaded into Lf NPs using this method.

#### 2.1.1. Sol-Oil or Water-in-Oil Emulsion Method: Anticancer Drug Delivery

Concerning anticancer drug delivery, one of the first applications of this method was to improve the delivery of doxorubicin (DOX). DOX is a highly effective chemotherapy drug used to treat various cancers, including leukemia as well as liver, breast, ovarian, and lung cancers; however, its clinical use is limited by its high toxicity to the heart and spleen and its poor oral absorption. To mitigate these drawbacks, sol-oil chemistry was employed to prepare DOX-loaded Lf NPs with a diameter of approximately 68 nm, an encapsulation efficiency (EE) of 79%, good physical stability (2.5–5% drug loss for incubation periods between 24 h and 90 days), and very low hemolytic activity [9,10].

Golla and coworkers demonstrated that the intravenous (i.v.) administration of DOX-loaded Lf NPs to rats with hepatocellular carcinoma (HCC) resulted in a significant reduction in the number of neoplastic liver nodules [9]. Moreover, the nanoformulation showed greater localization in the liver and enhanced bioavailability compared to free DOX, inducing minimal cardiotoxicity due to lower drug accumulation in the heart compared to DOX alone. In a subsequent study conducted by the same group, the oral administration of the same formulation to rats with HCC was also shown to significantly reduce the number of neoplastic liver nodules compared to both the positive control group (which received saline) and DOX alone [10]. Overall, both studies demonstrated that DOX-loaded Lf NPs exhibit superior efficacy, bioavailability (in a target-specific manner), and safety (the safety profile of DOX-loaded Lf NPs in the treated rats was comparable to that of untreated rats) compared to DOX for the treatment of HCC in rats.

An in vitro study by Ahmed and colleagues evaluated the potential of carboplatin (CBDCA)-loaded apotransferrin or Lf NPs (82–92 nm and 68–81 nm in size, and EE of 50% ± 2.3% and 52% ± 3.9%, zeta potential −23 ± 2 mV and −10 ± 3 mV, and polydispersity index, PI, 0.155 ± 0.5 and 0.340 ± 0.4, respectively) for the treatment of retinoblastoma, a common ocular malignancy in children [11]. In this study, the retinoblastoma cell line Y79 was used as the cellular model. Both types of CBDCA-loaded NPs are highly compatible and showed an enhanced internalization capacity into the target cells and increased antiproliferative activity compared to the free drug. The results suggested that this nanotechnology-based approach could significantly reduce the side effects of CBDCA while simultaneously improving its efficacy.

Although photosensitizer (PS)-mediated photodynamic therapy (PDT) is a treatment of choice for some cancers, its availability is limited due to the nature of PS. Effective PS are predominantly engineered on hydrophobic scaffolds, which limit their biodistribution and bioavailability and, in an aqueous environment, induce their aggregation, compromising the efficacy of PDT. Adimoolam and coworkers carried out a study in which Lf NPs were loaded with the photosensitizer Chlorin e6 (Ce6) to enhance its therapeutic potential [12]. These NPs had a size of approximately 80 nm, an EE of approximately 42%, and, once lyophilized, could be stored for a long time, showing efficient redispersion in aqueous media. Furthermore, over a 24 h period, Ce6 release from the NPs was higher at pH 5.5 (80%) than at neutral pH 7.4 (less than 20%). The most interesting result was that the encapsulation of Ce6 in NPs resulted in a 44-fold decrease in the amount of Ce6 required to induce light-mediated cell death in MDA-MB 231 and SK-OV-3 cells (human cancer cells representing breast and ovarian cancer, respectively) compared to free Ce6 [12].

Temozolomide (TMZ) is a chemotherapy drug used to treat aggressive brain tumors, such as glioblastoma multiforme (GBM) and anaplastic astrocytoma. It acts as an alkylating agent that damages the DNA of tumor cells, blocking their replication. One of the major challenges in cancer treatment is the development of resistance to chemotherapy. Achieving good therapeutic outcomes depends on the balance between optimal therapeutic TMZ doses and the side effects associated with dose escalation, which is limited by the risk of developing myelosuppressive disorders. Kumari and coworkers conducted a study in which TMZ was entrapped in Lf NPs with the aim of improving its therapeutic efficacy [13]. The NPs exhibited a size of about 70 nm, an EE of approximately 42%, and good physical stability at 4 °C. In vitro studies using the glioma cell line GL261 demonstrated that TMZ-loaded Lf NPs enhanced the uptake and internalization of TMZ into brain tumor tissue, compared to the free drug. Glioma-bearing C57BL/6 mice, in which tumors were developed by intracranial implantation of GL261 cells, were used for in vivo studies. These studies demonstrated that TMZ-loaded Lf NPs were nontoxic and had a better pharmacokinetic profile and brain accumulation than the free drug. Other important findings included reduced tumor volume/burden and a higher survival rate in glioma-bearing mice treated with TMZ-loaded Lf NPs. Overall, the results of this investigation confirm that Lf NPs are capable of crossing the BBB, resulting in improved active targeting of brain tumors [13].

In a subsequent study, Aurora Kinase B (AKB) small interfering (siRNA)-loaded Lf NPs (AKB–Lf NPs) were developed as an approach to sensitize the chemotherapeutic potential of TMZ. In particular, AKB-siRNA was used in combination with TMZ to prevent AKB gene expression [8]. The NPs ranged in size from 50 to 70 nm and exhibited an siRNA EE of approximately 56%. The PDI was 0.22 ± 0.1, indicating a narrow size distribution, and the zeta potential, ranging between −8 and −7 mV, was optimal for minimizing uptake by mononuclear macrophages and prolonging circulation time. Interestingly, after incubation with RNaseA for 1 h at 37 °C, the siRNA trapped in the Lf NPs remained intact, while the free siRNA was entirely degraded. Results from in vitro studies showed that AKB–Lf NPs alone or in combination with TMZ inhibited the viability of GL261 glioma cells by silencing the AKB gene and arresting the cell cycle. In vivo studies, using an orthotopic mouse model of GBM, demonstrated significant tumor growth inhibition and an approximately 2.5-fold increase in mouse survival when AKB–Lf NPs were injected i.v. alone or in combination with TMZ. The results of these studies suggest that siRNA-loaded Lf NPs could be used in combination with conventional chemotherapy to increase its efficacy, decrease its toxicity and, most importantly, reduce the emergence of resistance phenomena [8].

5-fluorouracil (5-FU) is an antineoplastic drug used to treat various types of solid tumors. To improve the antitumor efficacy of 5-FU, Kumari and Kondapi developed drug-loaded Lf NPs (5-FU-Lf NPs) [14]. These NPs were small in size (150 ± 20 nm) and had a high EE (64 ± 2.3%) and a very high drug loading (DL, 31.21 ± 2.5%). The PDI and zeta potential of the particles were 0.332 ± 0.1 and −2.5 ± 1 mV, respectively. These values, indicating a narrow size distribution and a surface charge of the NPs close to neutrality, suggest a stable dispersion in an aqueous medium. The in vitro study conducted on B16F10 melanoma cells showed that 5-FU-Lf NPs were stable, biocompatible, safe, and exhibited prolonged intracellular retention. In particular, 5-FU-Lf NPs demonstrated a high and specific release of 5-FU in melanoma cells, exerting a greater antitumor effect than free 5-FU. Therefore, the authors suggest Lf NPs as a delivery vehicle to enhance the antitumor effects of chemotherapeutic drugs such as 5-FU.

Chemotherapy with 5-FU and oxaliplatin (OX), used for the treatment of colon adenocarcinoma, has several contraindications, such as systemic toxicity and a narrow therapeutic index. To overcome these drawbacks, Ahmed and colleagues used sol-oil chemistry to obtain Lf NPs loaded with 5-FU and OX [15]. The 5-FU or OX-loaded Lf NPs had dimensions of 60–90 nm and 65–100 nm, respectively, and EE of approximately 48.6% (5-FU) and 51.3% (oxaliplatin). Moreover, 5-FU or OX-loaded Lf NPs had a zeta potential of −3.2 ± 1 and −2.3 ± 0.9 and a PDI of 0.170 ± 0.07 and 0.219 ± 0.09, respectively. The antiproliferative efficacy of Lf NPs loaded with 5-FU and OX was evaluated in vitro using the human colon adenocarcinoma cell line COLO-205, while pharmacokinetic and biodistribution analyses were performed in healthy rats, and efficacy and safety tests were carried out in rats with induced aberrant cryptic foci (ACF). In particular, the carcinogen azoxymethane (AOM) was utilized to induce ACFs, which subsequently transformed into neoplastic lesions in the colon of Wistar rats. Results from in vitro studies showed that 5-FU and OX-loaded Lf NPs exhibited greater antiproliferative activity than their drug-free counterparts. Results from in vivo experiments showed that, when drug-loaded NPs and the free drugs were injected i.v. into rats, drug-loaded NPs exhibited enhanced antitumor activity compared to the free drugs, along with lower systemic toxicity. Pharmacokinetic and biodistribution analyses performed in healthy rats also demonstrated that 5-FU and OX-loaded Lf NPs improved the pharmacokinetic profile and safety parameters of the drugs [15].

The alkylating agent carmustine (bis-chloroethylnitrosourea, BCNU)**,** due to its ability to cross the BBB, is used as a chemotherapy treatment regimen for various cancers, including glioblastoma. This antineoplastic drug can block the cell cycle and induce apoptosis. However, BCNU, being an extremely lipophilic drug, requires challenging conditions for complete solubilization and, more importantly, its use induces numerous adverse side effects. In a study conducted by Athmakur and collaborators, the therapeutic efficacy of BCNU-loaded Lf NPs was evaluated in vitro in the glioma cell line C6 [16]. These BCNU-loaded Lf NPs had a size less than 41 nm and an EE of approximately 43%. The small size of these NPs enhances their circulatory half-life and also makes them more efficient at crossing the BBB. DL in BCNU-loaded Lf NPs was 43 ± 3.7%, zeta potential value was −24.6 ± 5.94 mV and the PDI was 0.338 ± 0.05, indicating that the NPs are stable and exhibit a homogeneous narrow size distribution. Results of this study showed that drug release from the NPs was pH-dependent, being minimal at physiological and gastric pH levels and maximal at the pH of endocytic vesicles (pH 5.0) and the acidic extracellular pH of tumors, indicating reduced drug leakage during systemic circulation, lower systemic toxicity and enhanced tumor specificity. Finally, BCNU-loaded Lf NPs showed greater internalization capacity and antiproliferative activity in glioma cells, compared to the free drug.

Lf NPs also represent a safe alternative to viral vectors for gene delivery in oncology, leveraging receptor-mediated targeting and minimizing immunogenicity. The advantage of these NPs lies in their ability to overcome cellular and tissue barriers, allowing for efficient DNA delivery. In a study conducted by Kumari and Kondapi, the plasmid pGFPC1 (4.7 kb) encoding green fluorescent protein was used as a cargo gene to develop Lf NPs loaded with pDNA (pDNA-Lf NPs), which ranged in size around 140 ± 20 nm [17]. The zeta potential of pDNA-Lf NPs was −2 ± 0.1 mV and the PDI was 0.12; this slight negative surface charge of the NPs, by reducing opsonization by serum proteins, would promote a longer plasma half-life, while the low PI indicates the uniform size distribution of the NPs. The percentage of pDNA in Lf NPs was found to be approximately 58%. The use of pDNA-Lf NPs as a gene delivery vehicle was studied in human liver cancer (HepG2) and murine glioma (GL261) cells. The results obtained showed that pDNA-Lf NPs exhibited significant advantages, such as lack of cytotoxicity (even at the highest concentrations), resistance to nuclease digestion, biocompatibility, and stability during prolonged storage. This good stability could be related to both the strong electrostatic interaction between positively charged Lf and negatively charged DNA and to the presence of a DNA-binding domain in Lf that can further enhance the formation of a stable DNA-Lf nanocomplex [18]. Moreover, these NPs were able to target and facilitate gene uptake through receptor-mediated internalization in cell lines expressing the Lf receptor.

**Table 2:** Lactoferrin nanoparticles for antiviral drug delivery prepared by sol-oil or water-in-oil emulsion method.

Antiviral Drug Delivery			
Drug	Route of Administration	Major Outcomes	References
AZT	Wistar rats per os oral gavage	Increased efficacy, low organ-related toxicity, improved pharmacokinetic parameters, unchanged antiviral activity	[19]
EFV	Wistar rats per os oral gavage	Reduced toxicity, enhanced pharmacokinetic profile and doubled anti-HIV-1 activity compared to free EFV	[20]
ZDV/EFV/3TC	Wistar rats per os oral gavage	Reduced toxicity, increased anti-HIV-1 activity, pharmacokinetic profile and drug bioavailability compared to free drugs	[21]
EFV/CUR	Rats vaginal lavage	Absence of toxicity, improved pharmacokinetic profiles, anti-HIV-1 action	[22]
CUR/EFV CUR/DPV CUR/TDF	Wistar rats per os or intravaginal	The route of administration did not affect reproductive performance of rats or postnatal development of the offspring	[23]

AZT: Zidovudine; EFV: Efavirenz; ZDV: Zidovudine; 3TC: Lamivudine; CUR: curcumin; DPV: Dapivirine; TDF: Tenofovir.

#### 2.1.2. Sol-Oil or Water-in-Oil Emulsion Method: Antiviral Drug Delivery

Lf NPs prepared by the sol-oil emulsion method have also been used to fight viral infections, particularly HIV-1 infections.

In particular, over a decade ago, Lf NPs were used to encapsulate antiviral drugs such as zidovudine (AZT), the first approved antiretroviral drug for the treatment of AIDS. AZT is a potent antiviral drug with good bioavailability but several undesirable side effects. Furthermore, its amphiphilic nature results in low EE and significant drug loss when delivered via conventional liposomal vesicles. Encapsulation of AZT into Lf using the sol-oil method enabled to overcome this issue, yielding small NPs (in the 50–60 nm range) NPs, with good drug EE (67%) and good physical stability. AZT is released with biphasic kinetics: an initial rapid release (“burst”, 60%) when the particles are exposed to pH 5.0, followed over time by the release of 20% of the drug (80% of the loaded drug is released within 10 h). Oral administration of these NPs demonstrated improved pharmacokinetics (a 4-fold increase in the total drug concentration over time), a halving of organ toxicity, while maintaining unchanged antiviral activity [19]. These results strongly suggest that Lf NPs represent a safe formulation to improve antiretroviral drug delivery.

Subsequently, the same group used the sol-oil emulsion method to encapsulate efavirenz (EFV), a non-nucleoside reverse transcriptase inhibitor [20]. Although this drug is frequently used in the treatment of HIV-1 infection, its low bioavailability and high toxicity limit its use. To overcome these limitations, a formulation of EFV-loaded Lf NPs for oral administration has been developed [20]. These NPs had an average size of 45–60 nm and a 67% EE. The zeta potential of the EFV-loaded lactoferrin nanoparticles was −23 ± 1.2 mV, indicating colloidal stability, while the PDI of <0.341 indicated a homogeneous particle population with a narrow size distribution. In vitro and in vivo studies were conducted to establish the toxicity, stability, efficacy, biodistribution, and pharmacokinetics of EFV-loaded Lf NPs. Cell viability studies, conducted in peripheral blood mononuclear cells (PBMC), Jurkat T cells, and B16-F10 melanoma cells, demonstrated that EFV-loaded Lf NPs were at least 2-fold less toxic than free EFV. In vitro studies also demonstrated that EFV-loaded Lf NPs had an anti-HIV-1 activity two times greater than that of the free drug and that maximum drug release occurs at pH 5 and is minimal in both simulated gastric and intestinal fluids, confirming the stability of the NPs in the gastrointestinal environment and therefore their suitability for oral administration. Results from in vivo studies carried out in Wistar rats showed that EFV-loaded Lf NPs had enhanced oral bioavailability, significantly improved pharmacokinetic profile, and reduced toxicity compared to their free form [20].

In another study, the same investigators prepared Lf NPs loaded with EFV, zidovudine (ZDV) and lamivudine (3TC; 2′,3′-dideoxy-3′-thiacytidine) (named FLART-NPs) to improve the efficacy and bioavailability and reduce the toxicity of first-line antiretroviral therapy (FLART) [21]. This study was carried out by utilizing in vitro (T cell lymphoma SUP-T1, pro-monocytic U-937, and red blood cells) and in vivo (Wistar rats) models. FLART-NPs exhibited a mean diameter of 67 nm, a DL % of approximately 5%, and an EE > 58% (range 58 to 71%). The results of pH-dependent release studies showed that, for all drugs, the maximum amount of drug was released at pH 5.0, whereas only 20% was released after 24 h at pH 7.4. Triple-drug loaded NPs showed enhanced anti-HIV-1 activity, increased drug bioavailability, improved pharmacokinetic profile, and reduced drug-associated toxicity compared to the free drug combination. Therefore, the FLART nanoformulation makes a significant contribution to the field of innovative drug delivery systems, a contribution that warrants further investigation.

In a multiple prevention approach (MPT), the anti-HIV-1 drug EFV was loaded together with the antimicrobial-spermicidal agent curcumin (CUR) into Lf NPs (EC-Lf NPs) [22]. Since EFV and CUR are poorly soluble in water, encapsulating these two drugs in Lf helps address this issue, and EC-Lf NPs act as a broad-spectrum antimicrobial agent for HIV-1. As a matter of fact, EFV targets the HIV-1 reverse transcriptase enzyme, CUR contributes to the inhibition of viral replication by binding to the integrase and protease of HIV-1, and both CUR and Lf possess anti-inflammatory properties. The Lf NPs loaded with EFV and CUR had a size of approximately 40–70 nm with an EE of 61.5% ± 1.6% and 63 ± 1.9% for EFV and CUR, respectively. This EE was higher than that of single-drug NPs (EFV: 58.4 ± 1.79 and CUR: 59 ± 1.34%). Furthermore, EC-Lf NPs showed good physical stability when incubated in PBS at 4 °C and 25 °C. Results from the drug release studies showed that all three types of NPs, in single or combination form, showed the maximum release (>80%) at pH 5.0, a condition commonly associated with viral vaginal infections. In vivo experiments, performed on healthy female rats, demonstrated a significant increase in the amount of the delivered drugs, compared to free drugs, together with no negative effect on the vaginal ecosystem. EC-Lf NPs also showed improved pharmacokinetic profiles and reduced toxicity compared to free drugs, thus proving to be a safe and highly useful formulation.

Other studies have been conducted on Lf NPs loaded with CUR and various antiretroviral drugs. In particular, three types of dual-drug Lf NPs have been prepared: Lf NPs loaded with CUR/EFV, Lf NPs loaded with CUR/dapivirine (DPV), and Lf NPs loaded with CUR/tenofovir (TDF) [23]. Drug-loaded Lf NPs were administered to rats both vaginally (female rats) and orally (male and female rats). Vaginal administration had no effect on reproductive performance, fertility, or postnatal development, while oral administration caused a significant reduction in litter size without any influence on the developmental progression. The authors hypothesize that the reduction in litter size could be due to side effects of the utilized anti-HIV-1 drugs [23].

### 2.2. Thermal Denaturation (Heat Treatment) Method

**Figure 3:**
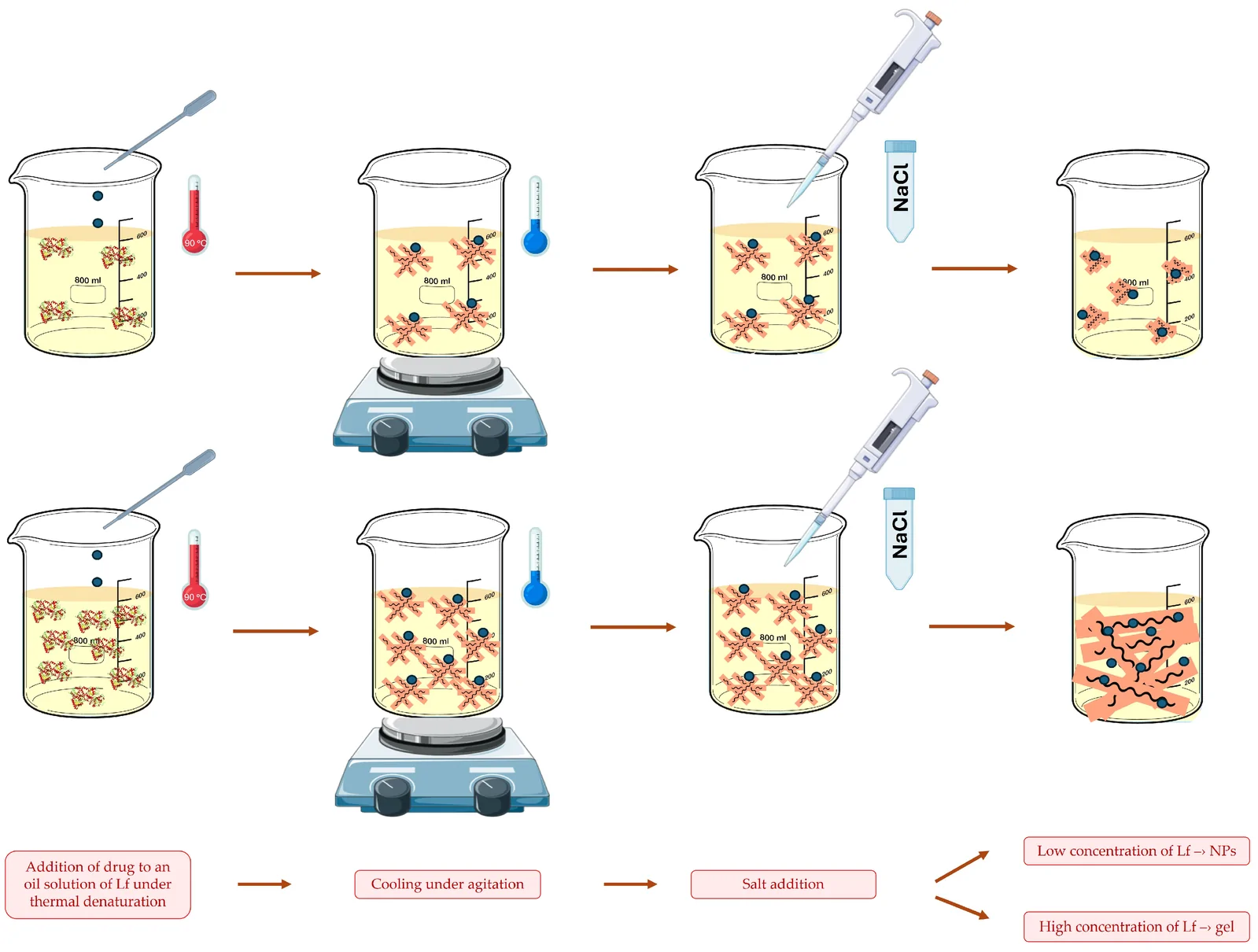
Preparation of lactoferrin nanoparticles by thermal denaturation method.

Because Lf, like other globular proteins, can form gels when exposed to specific pH or heat treatment, this protein has been used to encapsulate micronutrients, such as iron, for the food industry. Thermal gelation begins with a heating step to denature and polymerize the Lf, followed by a cooling step and the addition of an appropriate salt to induce protein aggregation (Table 3). The most commonly used salts include sodium chloride (NaCl), calcium chloride (CaCl_2_), magnesium chloride (MgCl_2_), magnesium sulfate (MgSO_4_), and ferric chloride (FeCl_3_) [24]. The Lf concentration used is a key factor influencing the process, as a high protein concentration leads to gel formation, while low concentrations result in NPs. Specifically, the Lf thermal denaturation method involves heating at 70–90 °C for 15–20 min in an oil bath, adding the drug solution dropwise, and finally cooling the mixture to room temperature under continuous stirring to allow the formation of drug-loaded Lf NPs [25] (Figure 3). Lf is relatively stable, and its thermal stability depends on its degree of iron saturation. Thermal denaturation of low iron-saturated or iron-free Lf (apo-Lf) typically begins above 60–70 °C, while iron-saturated Lf (holo-Lf) denatures between 85 and 93 °C. The N-lobe, which possesses a more open conformation compared to the C-lobe, is the first to undergo structural changes and denaturation. Thermal denaturation, causing the loss of the α-helical structure, leads to the exposure of hydrophobic groups, triggering the protein to aggregate. These Lf NPs prepared by thermal denaturation have high pH stability due to the strong hydrophobic bond between the protein macromolecules. NP stability is also enhanced by increased steric repulsion due to the presence of Lf glycan components extending into the aqueous medium. An important advantage of thermal denaturation is that this technique is generally considered environmentally friendly, as it requires neither organic solvents nor toxic cross-linking agents. In summary, while the sol-oil emulsion approach is a non-destructive encapsulation technique typically performed under mild physiological conditions without chemical cross-linkers or high temperatures, the thermal denaturation method applies intense thermal energy to intentionally unfold the protein backbone, exposing hydrophobic domains that drive subsequent aggregation into NPs.

Martins and colleagues developed and characterized Lf NPs as iron carriers; specifically, these Lf NPs were prepared by thermal gelation (75 °C, 20 min) to trap FeCl_3_ (35 mM) [24]. Regarding the influence of pH, it was observed that the NPs prepared at pH 7.0 had a diameter of 107–110 nm and an EE of approximately 53.8%, while NPs prepared at pH 4.0 had a diameter of approximately 383 nm. Moreover, NPs prepared at neutral pH had a zeta potential of + 22.1 ± 0.93 mV, a PDI of 0.218 ± 0.005 and demonstrated good stability to either temperature excursions (4–60 °C) or pH variations (pH 2.0–11.0) for a period of approximately 10 weeks. These characteristics suggest their potential use for iron release in food applications [24].

In a study by Li and co-workers, the antioxidant polyphenol cichoric acid (CA) was loaded into Lf matrices [26]. CA, present in many edible plants, is a phenolic compound with antioxidant, antiviral, antibacterial, and anti-inflammatory properties. However, this compound is highly unstable under certain conditions, such as aqueous or acidic environments, making its oral administration ineffective. Therefore, in this study, CA was encapsulated in Lf to increase therapeutic efficacy. Heating to 95 °C (neutral pH) yielded Lf NPs approximately 67 nm in diameter, with a zeta potential of +22.03 ± 1.18 mV and a PDI of 0.47 ± 0.02. These NPs exhibited excellent stability and had an EE of approximately 97.87–99.87% [26]. Differential scanning calorimetry thermograms showed that the encapsulation of CA in NPs also resulted in an increase in the thermal stability of Lf. Finally, in vitro studies based on the evaluation of 2,2-diphenyl-1-picryl-hydrazil (DPPH) radical scavenging and hydroxyl radical scavenging activity demonstrated that CA-loaded Lf NPs possessed greater antioxidant capacity than free CA [26].

Regarding in vivo studies, Mo and colleagues co-encapsulated simvastatin (SMV) and fenretinide (4-hydroxyphenylretinamide or 4-HPR) in Lf NPs modified with TPGS (D-α-tocopheryl polyethylene glycol succinate)-TAT (developed using TAT conjugated to TPGS) [25]. Both SMV, a statin used primarily to reduce LDL cholesterol and triglyceride levels, and 4-HPR, a synthetic retinoid derivative, have antitumor activity [27,28,29,30,31]. Despite the non-toxicity of both drugs, their application for the control of brain tumors is limited due to their poor ability to cross the BBB [25]. Therefore, to reach the central nervous system, the ability of Lf to cross the BBB was exploited. Briefly, the ethanolic solution of each drug was added dropwise to Lf in an aqueous solution, pre-stirred in an oil bath for 10 min at 80 °C. After removing the oil bath, the mixture was stirred until it reached room temperature. To improve intracerebral penetration, the penetrating peptide TAT was linked to TPGS to form an amphiphilic structure (TPGS-TAT). TPGS-TAT was finally linked to Lf NPs. TPGS-TAT Lf NPs exhibited a diameter of 177 nm, a zeta potential of +22 mV, good physical stability, and EE and DL capacities of approximately 52.8% and 1.53 ± 0.33% and 35.3% and 1.10 ± 0.44% for 4-HPR and SMV, respectively. Results from in vitro studies showed that dual-drug-loaded TPGS-TAT-Lf NPs exert a greater inhibitory effect on the U-87 human glioma cell line than the simple drug combination. In vivo studies utilizing two tumor-bearing mouse models (subcutaneous and orthotopic U-87 tumors) demonstrated that intravenous administration of dual-drug-loaded TPGS-TAT-Lf NPs had an enhanced anti-glioma effect in subcutaneous or orthotopic animal models of glioma compared to free or undecorated drug-loaded LF NPs [25]. These strategies, based on combination therapy and biomimetic delivery systems, could offer a new therapeutic option against glioma.

Regarding targets in the fight against cancer, it is necessary to consider the tumor immune microenvironment (TIME), which consists of various tumor components that suppress anti-tumor immunity, thereby promoting tumor immune evasion. Consequently, remodeling the TIME represents a strategic objective for achieving immunity. It has been suggested that the combined therapy of shikonin (SHK) and the thienotriazolodiazepine JQ1, by strengthening the immune normalization capacity, may be a good antitumor strategy [32]. SHK, a natural hydrophobic naphthoquinone isolated from the root of *Lithospermum erythrorhizon*, possesses antioxidant, anti-inflammatory, and antibacterial properties. Among its multiple pharmacological activities are ROS generation, inhibition of pyruvate kinase-M2, and downstream signaling molecules regulated by NF-κB [33]. It can also trigger antitumor immunity and suppress glucose metabolism in tumor cells, limiting tumor proliferation [34,35]. JQ1 is a thienotriazolodiazepine capable of inhibiting the bromodomain and the BET (Bromodomain and Extra-Terminal Domain) class of proteins. Specifically, by binding to the bromodomains of BET proteins, it blocks their interaction with acetylated histones and inhibits the expression of specific oncogenic genes. Based on these findings, Wang and collaborators prepared mannosylated Lf NPs (Man-Lf NPs) for the delivery of SHK and JQ1 via the mannose receptor and LRP-1, which are overexpressed in cancer cells and tumor-associated macrophages [36]. Man-Lf NPs had a diameter of approximately 150 nm with an EE and a DL capacity of 48.58% and 1.43 ± 0.26 and 85.76% and 2.69 ± 0.10 for SHK and JQ1, respectively. Furthermore, Man-Lf NPs were remarkably stable and exhibited a sustained in vitro drug release profile. Results from in vitro (the murine colon cancer cell line CT26 and the human umbilical vein endothelial cells HUVEC) and in vivo studies (immunocompetent CT26 tumor mouse model) demonstrated that Man (SHK/JQ1)-Lf NPs had a higher antitumor effect compared to both the free drug combination and dual-drug loaded Lf NPs [36]. Thus, the Man-Lf nanoformulation was able to influence several molecules of the immune microenvironment, confirming that the inhibition of tumor metabolism combined with the modulation of the immune response represents a therapeutic strategy of choice. 

**Table 3:** Lactoferrin nanoparticles prepared by thermal denaturation method.

Drug	Route of Administration	Major Outcomes	References
FeCl_3_	-	Good stability to temperature or pH variations	[24]
CA	-	Improved antioxidant potential compared to free CA	[26]
SMV/4-HPR	i.v. subcutaneous or orthotopic mouse models of glioma	Improved anti-glioma efficacy, compared to free drugs, in animal models of subcutaneous or orthotopic glioma	[25]
SHK/JQ1	i.v. immunocompetent CT26 tumor mouse model	Dual-drug-loaded mannosylated LF NPs have enhanced antitumor effect compared to either the free drug combination or dual-drug-loaded Lf NPs	[36]

FeCl_3_: ferric chloride; CA: cichoric acid; i.v.: intravenous; SMV: Simvastatin; 4-HPR: 4-hydroxyphenylretinamide or fenretinide; SHK: shikonin.

### 2.3. Desolvation Process

**Figure 4:**
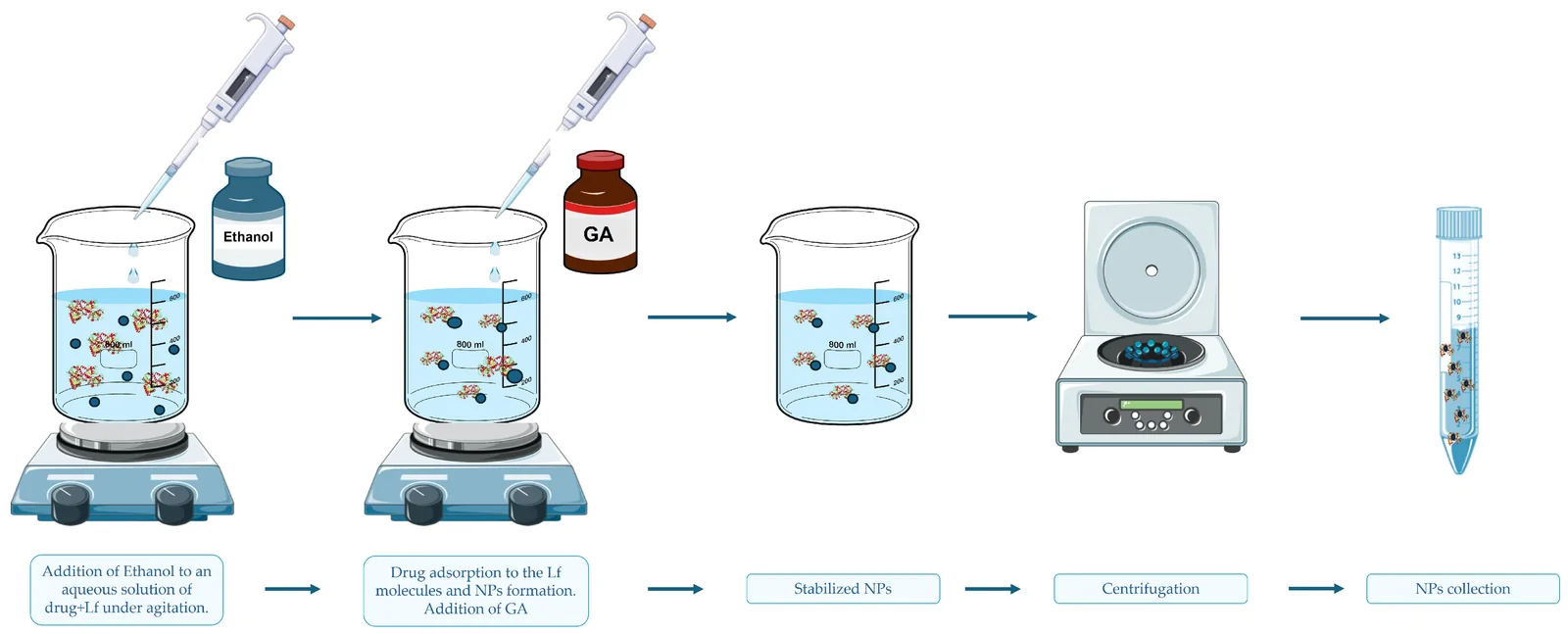
Preparation of lactoferrin nanoparticles by the desolvation technique.

Lf NPs can also be prepared using the desolvation technique (Table 4). For this procedure, a hydrophilic organic solvent (acting as a desolvating agent), such as acetone or ethanol, is gradually added to the water-dissolved Lf, leading to protein aggregation and thus to the formation of NPs. The NPs are then stabilized by adding a cross-linking agent, such as glutaraldehyde [2] (Figure 4).

Pandey and colleagues conducted a study to optimize the preparation of Lf NPs as CUR carriers (CUR-Lf NPs) using the desolvation technique and the Quality by Design (QbD) approach [37]. This technique allowed the production of Lf NPs with a diameter of approximately 190 nm, a yield of 75%, a PDI of +0.196 ± 0.004 and a zeta potential of +32.7 mV. Regarding EE, this was greater than 50% (53.7%), but this percentage can be increased by adjusting different parameters such as the sonication strength, temperature, cross-linking agent, pH, solvent, drug concentration, or drug/Lf ratio. No significant changes in the qualitative characteristics of the NPs were observed following loading with CUR. These results suggest that the proposed method can be used to load other drugs without significantly altering the quality of the loaded Lf NPs. Moreover, the increased antiproliferative activity of CUR-loaded Lf NPs compared to free CUR demonstrated that Lf NPs can be a good vehicle for the delivery of anticancer agents [37].

Subsequently, Taguchi and colleagues conducted a study to optimize the preparation conditions of glutaraldehyde-crosslinked Lf NPs using the desolvation technique [38]. The Lf NPs, 100–200 nm in size, were evaluated in vitro for their properties as carriers for oral and i.v. drug delivery. Specifically, NPs with a size of 150 nm were produced by adding 2-propanol as a desolvating solvent to the Lf solution adjusted to pH 6.0. These glutaraldehyde-crosslinked Lf NPs (PDI of 0.3 ±0.01 and zeta potential of about + 40 mV) were shown to be compatible with blood components (white blood cells (WBCs), red blood cells (RBCs), and platelets (PLTs)) and resistant to pepsin digestion. The study highlighted both the advantages of the desolvation method, particularly its cost-effectiveness and particle size control, as well as its disadvantages, such as the risk of protein denaturation during preparation. In conclusion, these NPs were suggested for i.v. and oral drug delivery.

Li and colleagues prepared CUR-loaded Lf NPs (CUR-Lf NPs) (84.8 ± 6.5 nm in diameter, PDI of 0.078 ± 0.016 and zeta potential of +22.8 ± 4.3 mV) using the desolvation method to develop a nasal-to-brain delivery system and evaluate their neuroprotective effects in vitro and brain accumulation in vivo [39]. CUR-Lf NPs showed EE and DL values of 91.2% ± 3.6% and 9.6% ± 0.8%, respectively. Results from in vitro studies showed that the permeability coefficient of the CUR-Lf NPs in MDCK (Madin-Darby canine kidney) cells (a widely used in vitro model for evaluating passive permeability and transporter-mediated efflux) was 50-fold higher than that of CUR suspension alone, suggesting that the NPs could improve the absorption efficiency of CUR in the nasal cavity. CUR-Lf NPs also provided good protection against Aβ25-35-induced nerve damage in PC12 cells (an established neuronal cell model). Regarding in vivo pharmacokinetic studies, the brain targeting efficiency of CUR-Lf NPs after intranasal administration in Sprague-Dawley rats was observed to be 248.1%, and the percentage of direct nasal-to-brain transport was 59.7%. Brain targeting with intranasal administration was superior to that with i.v. administration. In conclusion, nasal-to-brain delivery of CUR-Lf NPs was shown to be a promising strategy for neuroprotection.

### 2.4. Nanoparticle Albumin-Bound (Nab) Technique

Because bovine Lf is rich in cysteine, with 17 disulfide bonds, it has a strong tendency to form NPs via modified nanoparticle albumin-bound (Nab) technology (Table 5). This process safely encapsulates hydrophobic drugs without toxic surfactants using high-pressure homogenization, lipid-aqueous phase emulsification, and solvent evaporation.

**Figure 5:**
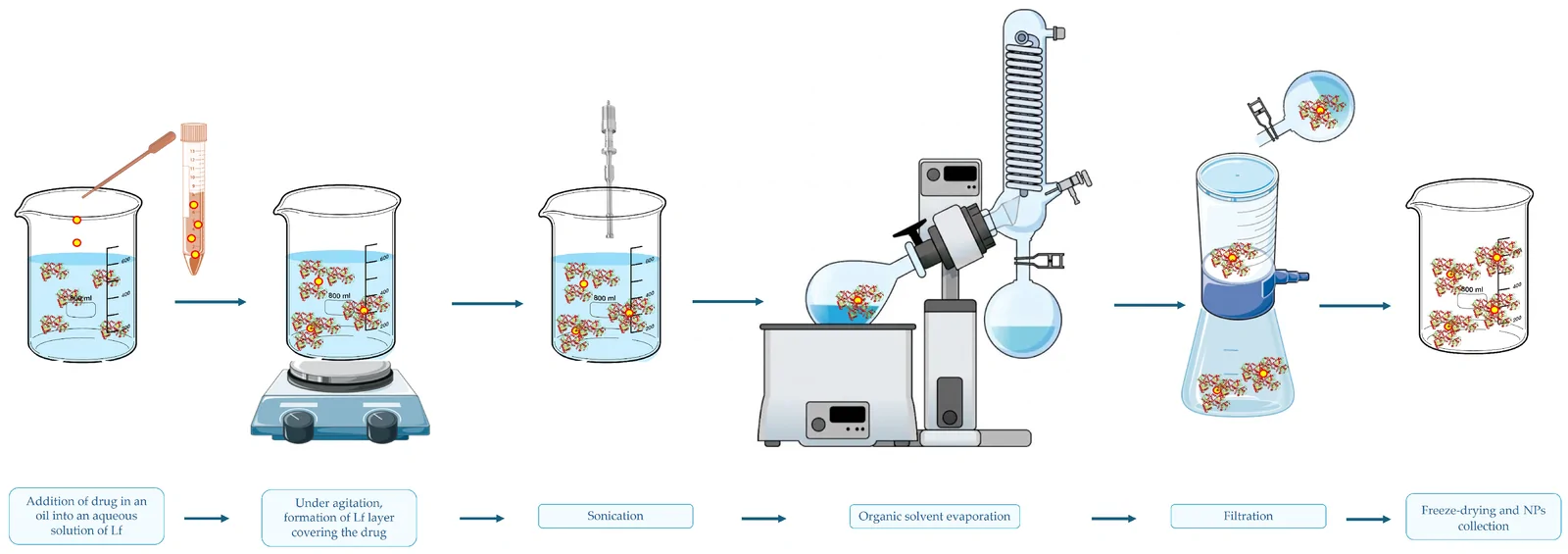
Preparation of lactoferrin nanoparticles by Nab technique.

Nab technology involves the slow addition of a drug in a hydrophobic solvent (oil phase) to Lf in a hydrophilic solvent (aqueous phase). The mixture is then homogenized at low speed, leading to the formation of a protein layer covering the drug particles, and is subsequently subjected to high-energy sonication. The final nanosuspension, obtained after removing the organic solvent via a rotary evaporator, is then filtered and freeze-dried (Figure 5). The preparation of drug-loaded Lf NPs by this method strictly depends on the drug/Lf ratio, the pH of the medium and the potency and duration of homogenization [40]. Using Nab technology, both oleanolic acid (OA) and gambogic acid (GA) were loaded onto Lf NPs with sizes of approximately 100–200 nm [40,41].

GA, which has anti-inflammatory, antioxidant, antitumor, and neuroprotective properties, is the major active component of *Garcinia hanburyi*. Since GA is poorly hydrophilic, it must be conjugated with L-arginine or solubilized in Cremophor EL (CrEL), leading to adverse effects such as allergies and toxicity. To overcome these disadvantages, an oral nanoformulation of Lf/GA was developed using Nab technology [41]. These GA-loaded Lf NPs exhibited a size of about 150 nm, EE of 92.3 ± 7.2%, DL of 9.04 ± 0.7, zeta potential of + 20 mV, and showed good stability and slow release in in vitro systems. As a matter of fact, slow release of GA was observed after 24 h in both simulated gastric and intestinal fluid (8.74% and 15.4%, respectively). GA and GA-loaded Lf NPs intestinal absorption mechanism was studied in vivo by single-pass in situ perfusion (duodenum, jejunum, ileum, and colon) in Wistar rats. The antitumor activity of GA-loaded Lf NPs and GA against S180 solid tumors was studied in vivo using ICR (Institute of Cancer Research) mice. These mice had previously been injected subcutaneously with a suspension of S180 cells (a murine Sarcoma cancer cell line). Concerning the antitumor activity, it was observed that oral administration of GA-loaded Lf NPs had a better antitumor effect in mice than intravenous injection of arginine-conjugated GA. These results demonstrated that Lf NPs increase the oral absorption of hydrophobic drugs and reduce their toxicity [41]. 

Using the same technology, oleanolic acid (OA) loaded Lf NPs were prepared [40]. OA, a pentacyclic triterpenoid widely distributed in the plant kingdom [42], possesses anti-inflammatory, antioxidant, antitumor, and hepatoprotective activities [43]; however, similar to GA, its therapeutic efficacy has been limited due to poor aqueous solubility and bioavailability [44]. The OA-loaded Lf NPs prepared with Nab technology exhibited a size of about 202 nm, a zeta potential of +27.1 ± 0.3 mV, a PDI of 0.150 ± 0.032, as well as a high percentage EE and DL (92.59% ± 3.24% and 12.44% ±2.52%, respectively). Studies conducted in Sprague-Dawley rats demonstrated that oral administration of these NPs resulted in a significant improvement in the bioavailability and pharmacokinetic profile of OA compared to its free form [40].

Recently, a modified Nab method has also been successfully used in the field of infectious diseases [45]. This approach is extremely important as the treatment of microbial infections has become increasingly complex due to the development of resistant strains and the intracellular localization of many pathogens. In this study, carried out by Andima and co-workers, modified Nab technology was used to load Lf NPs with a combination of two drugs: natural alkaloids (berberine or sanguinarine) and vancomycin or imipenem [45]. In particular, Lf NPs were loaded with sanguinarine (S) and imipenem (I), sanguinarine and vancomycin (V), berberine (B) and I, B and V and labeled as Lf-SI, Lf-SV, Lf-BI, and Lf-BV, respectively. These Lf NPs were prepared by a modified Nab method, whereby mPEG2000 was included to stabilize the NPs in solution. NPs showed a high EE with an average of 82.3% and a DL capacity of about 1%, had an average size of 191.8 ± 11.1 nm, a uniform size distribution (PDI 0.23 ± 0.04), and a positive zeta potential (+8.03 ± 3.84 mV). The NPs showed a gradual drug release at physiological pH over a 24 h period. Drug release reached a plateau after 6 h, followed by a slower release phase; within 24 h, approximately 98% of I, 50% of S, 95% of V, and 40% of B had been released. The hallmark of this study was the exploitation of the pathogen-host relationship in the Lf-mediated “war for iron” to target intracellular bacteria with a dual combination of antibiotics loaded into Lf NPs. The results demonstrated that the drug-loaded Lf NPs were able to effectively eliminate intracellular *Staphylococcus aureus* (Newman strain) compared to free drug combinations. Overall, the study demonstrated the ability of Lf NPs to deliver anti-infective agents for treating hard-to-eradicate intracellular bacteria.

**Table 5:** Lactoferrin nanoparticles prepared by the albumin-bound method.

Drug	Route of Administration	Major Outcomes	References
GA	Per os ICR mice	Increased antitumor effect in sarcoma mice compared to i.v. administration of arginine-conjugated GA	[41]
OA	Per os Sprague-Dawley rats	Increased bioavailability of OA in Sprague Dawley rats	[40]
S, B, I, V	In vitro *S. aureus* “Newman” THP-1 and A549 cells	Effective administration of anti-infective agents capable of treating difficult-to-eliminate intracellular bacteria.	[45]

GA: Gambogic acid; per os: oral; i.v.: intravenous; OA: oleanolic acid; S: Sanguinarine; B: berberine; I: imipenem; V: vancomycin; THP-1: human leukemia monocytic cell line; A549: human lung adenocarcinoma basal epithelial cells.

### 2.5. Electrostatic Complexation: Combination of Oppositely Charged Molecules

**Figure 6:**
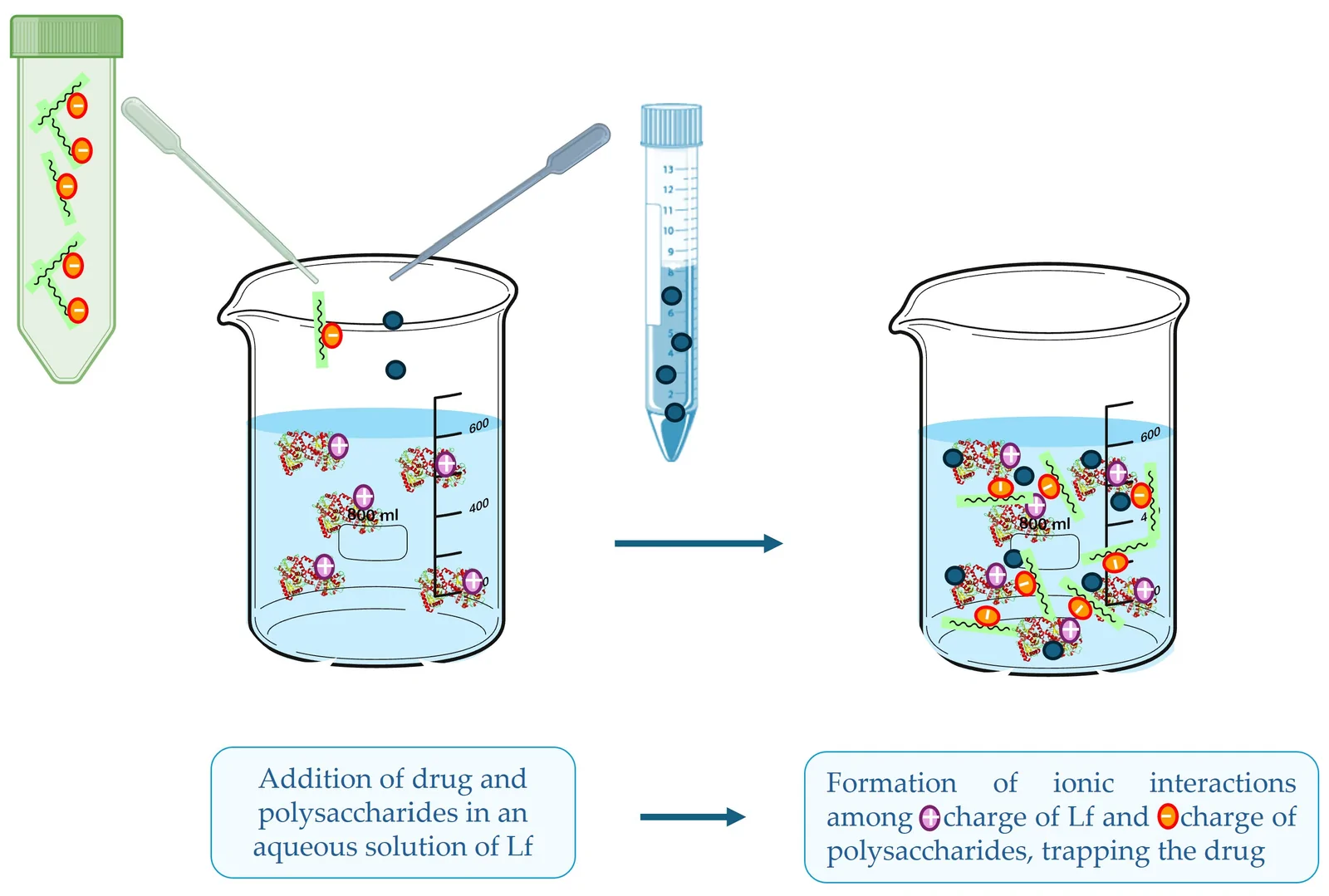
Preparation of lactoferrin nanoparticles by electrostatic complexation.

The formation of complexes between positively charged proteins and other negatively charged compounds, such as polysaccharides, has enabled the development of delivery systems used in various fields, including pharmaceuticals [46] (Table 6). Because Lf is a cationic protein with a high isoelectric point (pI: 8.0 to 8.7), it can form stable polyelectrolyte nanocomplexes through electrostatic interactions with anionic polysaccharides or proteins. In particular, these electrostatic interactions occur between the positive charges present in the N-terminal region of Lf and the negative carboxyl groups present on the anionic polysaccharide (Figure 6). Furthermore, it has been observed that the addition of a third component, such as chitosan or phenolic compounds, results in an increase in the stability of the nanocomplex [46].

In a study by Peinado and co-workers, Lf NPs with a diameter of 200–400 nm (zeta potential—20–40 mV) were obtained by electrostatic complexation of thermally denatured Lf particles with anionic polysaccharides (alginate, carrageenan, or pectin) [47]. Specifically, Lf NPs (diameter 200–400 nm, isoelectric point approximately 8.5) were obtained by heat treatment (91 °C, pH 7.0) of a 0.2% Lf solution. It was observed that Lf-pectin complexes were more stable to the addition of salt, compared to Lf-carrageenan and Lf-alginate complexes. This superior salt tolerance is driven by pectin’s structural architecture. The highly charged, linear chains of alginate and carrageenan cause immediate salt-induced charge screening and aggregation, while the highly branched, neutral sugar side chains of pectin provide robust steric hindrance, increasing NP stability when electrostatic forces are neutralized by salts.

These results could be exploited for the application of Lf-polysaccharide nanocomplexes as functional components in the pharmaceutical or food sectors.

In a subsequent study by Bengoechea and colleagues, the interaction of Lf with a high-methoxy pectin was investigated in aqueous solutions, at various temperatures (30–90 °C) and pH values [46]. In the pH range from 7.75 to 5, the particle size remained constant (110.6 ± 10.2 nm), whereas as the pH decreased, the particle size increased (360 ± 70 nm at pH 3.9). At neutral pH, increasing the amount of pectin added to the aqueous Lf solution changed the electrical charge of the solution from positive to negative, indicating the formation of an electrostatic complex. It is important to note that during heating, the stability of these complexes to aggregation was much higher than that of Lf alone. Because Lf is heat-stable when part of an electrostatic complex, the electrostatic nanocomplexes obtained in this study represent a suitable means to incorporate Lf into foods and exploit its beneficial functional properties [46]. 

Another study was conducted to establish the optimal weight ratios for the formation of polyelectrolyte complexes based on Lf, N-succinyl chitosan and galactomannan. Lf/N-succinyl chitosan-galactomannan nanocomplexes were formed by simple mixing (in 0.15 M NaCl, pH 6.5 ± 0.1) in ratios of 1:2:3 and 1:3:2 before heat treatment. Heat treatment below the protein denaturation temperature resulted in the formation of spherical particles measuring 250–400 nm with an Lf content of 76–87, a zeta potential of −12 to −17 mV, and good storage stability [48].

Yan et al. produced polyelectrolyte complex NPs (PEC NPs) through electrostatic interactions between heat-denatured, positively charged Lf particles and negatively charged pectin in a 1:1 (*w*/*w*) mass ratio at pH 4.5 [49]. These NPs, with a size of ∼208 nm, were used as CUR carriers. The hydrophobic CUR was encapsulated with high efficiency (∼85.3%), and a controlled, pH-dependent release (greater at acidic pH) was observed in vitro. Regarding the influence of pH, PDI values decreased from 0.8 to 0.3 as the pH increased from 2.0 to 4.0, and then gradually increased from 0.3 to 0.5 as the pH rose from 4.0 to 7.0. The negative zeta potential of the NPs increased from −19.5 mV to −32.7 mV as the LF/pectin mass ratio varied from 3:1 to 1:3, whereas it remained constant as the LF/pectin mass ratio varied from 1:3 to 1:1. Interestingly, encapsulated CUR showed improved water solubility and antioxidant capacity compared to the free substance, through its ability to scavenge 2,2-diphenyl-1-picrylhydrazyl (DPPH) radicals. The results of this research indicate that Lf-PEC NPs are efficient carriers for the delivery of hydrophobic CUR and suggest potential applications in the food and pharmaceutical sectors.

It has also been observed that Lf can form electrostatic nanocomplexes with anionic polysaccharides without heating. Abd El-Waki and co-workers developed Lf-chondroitin sulfate (ChS) nanocomplexes for the combined administration of DOX and ellagic acid (EA) nanocrystals to lung cancer cells by exploiting the electrostatic interaction between the positively charged Lf and the negatively charged ChS [50]. In this work, EA, a plant-derived drug [50], was co-encapsulated with the cytotoxic drug DOX in hydrophilic Lf-ChS NPs, and the nanocomplex was then converted into inhalable nanocomposites (with a size of 192.3 nm, a PDI of 0.185 ± 0.089 and a zeta potential of −26.9 ± 0.9) by spray drying. The nanocomplex (EE of 91.6% and 96.26% for DOX and EA, respectively) showed a faster sequential release of EA, followed by DOX, and increased cytotoxicity and internalization in A549 lung cancer cells. The antitumor efficacy of EA/DOX-loaded NPs was evaluated and compared to free EA/DOX solution in BALB/c mice. To induce lung cancer, mice were given an intraperitoneal (i.p.) injection of 1.5 g/kg of urethane, followed by a booster dose at week 8. An insufflator was used to administer drug-loaded NPs or drugs alone directly into the lungs of mice. The developed dual-drug-loaded inhalable NPs demonstrated greater antitumor efficacy than free inhalable combined drugs in lung tumor-bearing mice [50].

The formation of NPs through electrostatic complexation has recently been applied in the fight against infectious diseases. Lf NPs and gellan gum (G, a negatively charged, biodegradable, non-toxic exopolysaccharide) were produced to enhance the antimicrobial properties of Lf [51]. The antimicrobial activity of G/Lf NPs was studied against *Staphylococcus aureus* (Gram-positive) and *Escherichia coli* (Gram-negative). Surface charge and size were observed to be key parameters for antibacterial efficacy. In particular, NPs with a 9Lf:1G mass ratio were the only samples to exhibit bacteriostatic activity. Indeed, these NPs exhibited a zeta potential of +21.20 ± 1.25 mV, indicating that the G amount was insufficient to neutralize all the amino groups of the Lf, and suggesting potential antimicrobial activity associated with the cationic nature of the Lf. The high density of positive surface charges induces electrostatic repulsion among the NPs, resulting in the formation of NPs smaller than 100 nm (92.03 nm) with low polydispersity. These NPs demonstrated a minimum inhibitory concentration against *Staphylococcus aureus* six times lower (0.3 mg/mL) than pure Lf (2 mg/mL), although their bacteriostatic action was inhibited in the presence of divalent cations. The G/Lf NPs were also shown to extend the shelf life of fruit by up to 6 days in the presence of carboxymethylcellulose. Therefore, the G/Lf NPs proved to be a promising tool for enhancing the antimicrobial activity of Lf and expanding its application as a food preservative.

### 2.6. Amphiphilic Lf Micelles

**Figure 7:**
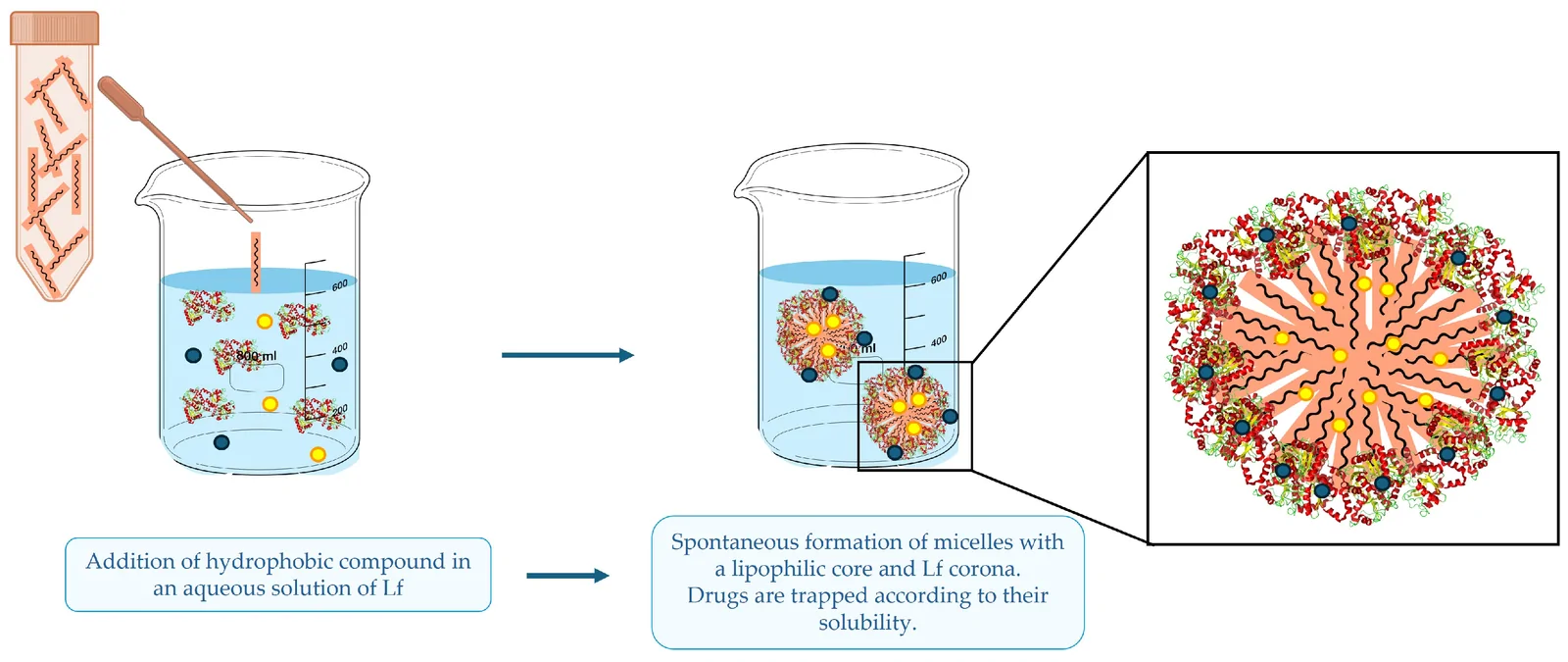
Preparation of amphiphilic lactoferrin micelles.

Amphiphilic molecules can self-assemble in an aqueous environment to form micelles with an outward-facing hydrophilic corona and an inward-facing hydrophobic core. Micelles, based on their high physical stability and prolonged circulation compared to other types of nanocarriers, are widely used in drug delivery [52] (Table 7). In particular, Lf has been used for the fabrication of micelles due to its low immunogenicity, high biocompatibility, and potential for active targeting [53,54] (Figure 7). 

Specifically, an amphiphilic copolymer was fabricated from the hydrophilic protein Lf and the hydrophobic zein (corn protein) using the 1-(3-Dimethylaminopropyl)-3-ethyl carbodiimide hydrochloride/N-hydroxysuccinimide (EDC/NHS) carbodiimide reaction, a widely used, water-soluble method for conjugating carboxyl groups to primary amines to form stable amide bonds [53,54]. Subsequently, rapamycin (RAP) and wogonin (WOG) were trapped in the micelles, followed by cross-linking with 8% glutaraldehyde. The cross-linked micelles had a hydrodynamic size of approximately 212 nm, a PDI of 0.206 ± 0.02 and a zeta potential of + 25.9 ± 4.26. Concerning RAP, EE and DL percentages were 61.90 ± 3.10 and 5.83 ± 0.29, respectively, while for WOG, they were 98.00 ± 4.90 and 4.67 ± 0.23, respectively. These stable biocompatible amphiphilic polymeric micelles exhibited increased in vitro toxicity against the MCF-7 human breast cancer cell line. Cellular uptake was facilitated by the active targeting of Lf to the cancer cell surface. An in vivo antitumor efficacy study using the Ehrlich ascites tumor (EAT) mouse model demonstrated an enhanced antitumor effect for these dual-loaded cross-linked micelles [53]. This enhanced efficacy is due to micellar stability mediated by cross-linking, improved targeting capability, and, consequently, enhanced accumulation in tumor tissues. 

The same amphiphilic micelles were used to fabricate magnetic micelles loaded with dasatinib (DAS) and oleic acid (OA)-coated magnetic nanoparticles, both co-encapsulated within the hydrophobic zein core by evaporation/sonication. These micelles (size 232.4 ± 20.33 nm, PDI 0.259 ± 0.02, zeta potential +41.93 ± 3.34 mV, EE 83.01 ± 4.98%, DL 7.42 ± 0.47%) showed enhanced in vitro toxicity against the MDA-MB-231 human breast cancer cell line. Furthermore, the micelles exhibited greater physical stability and superior sustained drug release, and inhibited cell migration, proving to be promising nanocarriers for magnetically guided drug delivery [54]. These results are extremely interesting since the zein-Lf copolymer is a GRAS (Generally Recognized As Safe) material, composed of natural protein components and has much lower cost and toxicity than the commonly used synthetic amphiphilic copolymers [55]. 

In another study, zein-Lf micelles containing DAS were encapsulated in sodium alginate spheroids via an on-chip microfluidic method. These charged micelles had a hydrodynamic diameter of 293 ± 10.30 nm, a zeta potential of approximately + 34.69 mV, a PDI of 1.3 and an encapsulation efficiency of 83.52 ± 3.26%. These spheroids showed sustained drug release with enhanced in vitro toxic effect against the human breast cancer cell line MDA-MB-231 [56].

Lf has also been used to develop micelles to be administered to alleviate or treat Alzheimer’s disease (AD). Specifically, Lf was covalently linked to conjugated linoleic acid (CLA) via an EDC coupling reaction, and the CLA was then loaded into the hydrophobic core of the micelles via solvent evaporation [57]. CLA-based micelles exhibited a size of approximately 53 nm (PDI of 0.301± 0.011, zeta potential of -32.8± 3.66 mV, EE about 76.0%, DL about 8.7%) with sustained in vitro drug release at pH 6.8 and improved hemocompatibility [57]. In vivo studies in Wistar rats with aluminum chloride (AlCl_3_)-induced AD revealed improved BBB crossing of these micelles, along with improved cognitive performance and reduced oxidative stress and inflammation. Moreover, a greater anti-AD effect was observed for Lf-CLA micelles compared to the Lf-CLA blend. Therefore, LF-CLA micelles appear to represent a safe, actively targeted nanotechnology platform capable of counteracting AD [57].

**Table 7:** Lactoferrin-drug amphiphilic micelles.

Drug	Route of Administration	Major Outcomes	References
RAP/WOG, Lf and zein conjugated	In vitro: MCF-7 human breast cancer cells In vivo: i.v., EAT mice	Enhanced antitumor activity of the combined drug nano-delivery compared with the free drug combination.	[53]
DAS/OA oleic acid-coated magnetite NPs, Lf and zein conjugated	In vitro: MDA-MB-231 breast cancer cells	Increased toxicity, reduced cell migration in the presence of an external magnetic field.	[54]
DAS, Lf and zein conjugated	In vitro: MDA-MB-231 breast cancer cells	Increased in vitro toxicity and sustained drug release	[56]
Conjugated CLA	In vivo: per os AlCl_3_ AD-induced Wistar rats	Reduction in AD symptoms, greater anti-AD effect of Lf-CLA micelles compared to Lf-CLA blend	[57]

RAP: rapamycin; WOG: wogonin; i.v.: intravenous; EAT: Ehrlich Ascites Tumor; DAS: dasatininb; OA: oleic acid; CLA: conjugated linoleic acid; per os: oral; AlCl_3_: aluminum chloride; AD: Alzheimer disease.

### 2.7. Lactoferrin-Hydrophobic Moieties (or Drug) Nanoconjugates: Self-Assembly NPs

**Figure 8:**
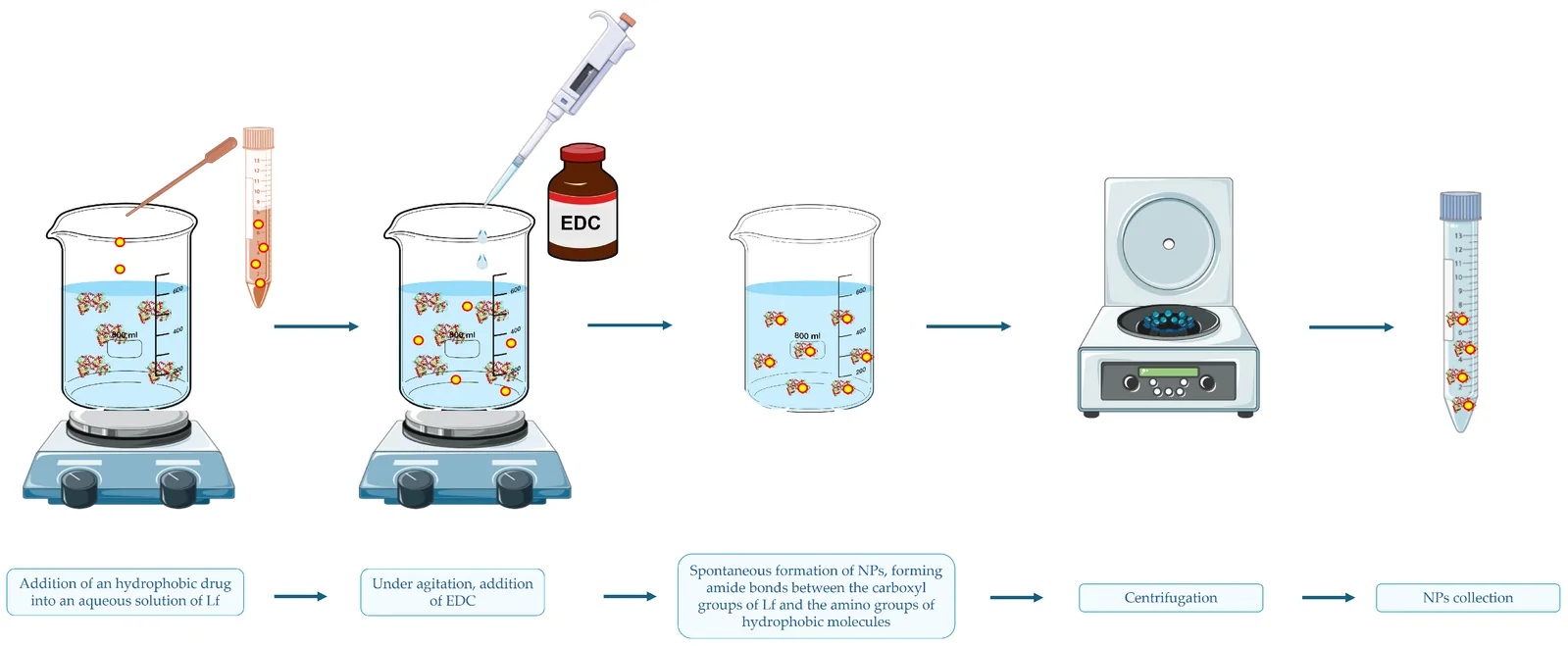
Preparation of self-assembled nanoparticles.

As we have already pointed out, Lf, due to its hydrophilic properties, can be chemically conjugated with various hydrophobic molecules or drugs (Table 8). This conjugation promotes self-assembly, leading to the formation of water-soluble NPs. Similarly, cross-linking agents such as EDC (1-Ethyl-3-(3-dimethylaminopropyl)carbodiimide) are often used to form amide bonds between the carboxyl groups of Lf and the amino groups of hydrophobic molecules. This carbodiimide-mediated coupling reaction drives the self-assembly of Lf into stable nanoformulations (Figure 8). 

Kamalinia and co-workers carried out a study in which an iron chelator, deferasirox (DFX)**,** was conjugated to Lf via a carbodiimide-mediated coupling reaction to create a novel drug delivery system with increased brain permeability through receptor-mediated transcytosis [58]. Water-soluble nanostructures (with sizes ranging from 100 to 500 nm, PDI from 0.310 ± 0.051 to 0.459 ± 0.034 and zeta potential from −8.830 ± 2.753 mV to + 1.477 ± 0.092 mV) were obtained. Size, PDI, and zeta potential of the nanostructures were correlated with the molar excess of deferasirox. With a larger excess, the size decreased from 460 nm to 137 nm, the PDI dropped from approximately 0.46 to 0.31, and the zeta potential shifted from negative (−8.83 mV) to positive (+1.45 mV). The neuroprotective effects of NPs, together with the related molecular mechanisms involved, were evaluated in the rat pheochromocytoma cell line (PC12). NPs size and surface charge played a fundamental role in the efficiency of cellular internalization and targeted drug delivery. The highest levels of neuroprotection were achieved with NPs 137 nm in diameter, likely because the small size of these conjugated nanostructures facilitated cellular penetration. Furthermore, positively charged nanoparticles promoted more effective cellular internalization due to their higher affinity for the cell membrane. In vivo studies were also conducted, which demonstrated that i.p. injection of these 137 nm NPs significantly attenuated learning deficits induced by beta-amyloid injection in a Wistar rat model of AD. These results demonstrated the neuroprotective effect of DFX conjugated to Lf in the management of neurodegenerative disorders through metal chelation therapy.

Chaharband et al. conducted a study in which CUR was conjugated with Lf [59]. Specifically, the Lf carboxyl moiety was activated via a carbodiimide reagent and coupled to the CUR hydroxyl group, forming an ester bond. NPs (diameter of approximately 165 ± 26.15 nm and PDI 0.308 ± 0.045) with significantly improved cytotoxicity against the human colorectal carcinoma cell line HCT116 were obtained. These CUR-Lf NPs not only solved the problem of CUR insolubility but, thanks to passive targeting, showed high cellular uptake and, more importantly, greater efficacy than free CUR.

A Lf-DOX conjugate was also synthesized via a carbodiimide coupling reaction, which forms an amide bond through the activation of the carboxyl group of Lf and its binding to the amino group of DOX [60]. Both apo-Lf-DOX and holo-Lf-DOX showed increased cytotoxicity in DU145 human prostate cancer cells compared to free DOX. Specifically, holo-Lf-DOX induced significant apoptosis and greater chemosensitivity than apo-Lf-DOX and was also effective in drug-resistant ADR1000-DU145 cells. In vivo studies were also conducted using a transgenic mouse prostate adenocarcinoma (TRAMP) model that faithfully reproduces the pathogenesis of human prostate cancer. Interestingly, treatment of TRAMP mice with oral administration of holo-Lf-DOX inhibited tumor development, prolonged survival, reduced DOX-induced toxicity, and increased RBC and serum levels of the anticancer molecules TNF-α, IFN-γ, CCL4, and CCL17. The results obtained suggest that targeted oral nanoformulations could be used to deliver active compounds against prostate cancer [60].

The emergence of drug resistance represents a significant risk in breast cancer treatment. One approach to overcoming this obstacle is combination therapy, which involves the simultaneous use of drugs acting on different targets. In a study by Ali and co-workers, Lf-conjugated mesoporous silica NPs (MSNPs) were developed for the combined delivery of the cytotoxic drug pemetrexed (PMT, an antifolate chemotherapy drug) and the phytopharmaceutical EA for synergistic breast cancer therapy [61]. Specifically, hydrophobic EA was encapsulated in MSNPs modified with positively charged amino groups, while water-soluble PMT was chemically anchored to the surface of Lf-coated MSNPs by reaction with carbodiimide to prevent premature drug release and systemic toxicity. Dual-drug-loaded Lf-MSNPs (284 nm diameter, PDI 0.226, zeta potential + 15.8 ± 0.27 mV, EE of EA 86.1 ± 0.84%, conjugation efficiency of PMT 63.7 ± 0.45%, DL 39 ± 0.61% and 16 ± 0.22% for EA and PMT, respectively) demonstrated faster sequential release of EA followed by sustained release of PMT and increased internalization (via Lf receptor-mediated endocytosis) and cytotoxicity against MCF-7 (Michigan Cancer Foundation-7) breast cancer cells compared to free drugs. Synergy between the two loaded drugs was also observed. Moreover, as with other Lf-conjugated NPs, high cellular uptake of the nanocarriers was observed via Lf receptor-mediated endocytosis.

This technology that exploits the Lf self-assembly has also found application in the field of infectious diseases, mainly in the fight against intracellular bacteria. In particular, in a study conducted by Wang and colleagues, protein self-assembly technology was used to load Lf NPs (Rif@Lf NPs) with the antibiotic rifampicin [62]. The Rif@Lf NPs had a diameter of 123.6 nm, a zeta potential of +5.3 mV in PBS 7.4, and a PDI of 0.161. The EE and DL of Rif in Rif@Lf NPs were 28.9% and 2.45%, respectively. Moreover, Rif@Lf NPs demonstrated remarkable stability at both 4 °C and 37 °C. Compared to free RIF or Rif@BSA NPs, Rif@Lf NPs were observed to specifically target bacteria-infected RAW 264.7 murine macrophages, increasing the accumulation of intracellular RIF and specifically adhering to bacteria within macrophages. Indeed, at pH 6.0 (a condition simulating the acidic microenvironment of infected macrophages), the zeta potential of Rif@Lf NPs shifts towards a higher positive charge (+14.1 mV), favoring adhesion to intracellular bacteria. Specifically, in a redox intracellular environment, Rif@Lf NPs disassemble to release RIF. In this scenario, Lf NPs exploit receptor-mediated endocytosis (via LfR/LRP1) to actively cross the host cell membrane; Lf physically destabilizes the bacterial cell wall; and the acidic microenvironment of the infected cells triggers a smart, stimuli-responsive disassembly of the NPs, releasing a localized burst of RIF directly at the infection site. In vivo studies have demonstrated that, compared to free Rif and Rif@BSA NPs, Rif@Lf NPs exhibit significant therapeutic effects in various disease models, including subcutaneous infection, systemic inflammation, sepsis, and bacterial keratitis. Furthermore, these NPs demonstrate a favorable safety profile in mice. By combining the antibacterial activity of Lf with that of RIF, Rif@Lf NPs demonstrated broad-spectrum antibiotic capabilities against intracellular bacteria in vitro and in vivo and against biofilm formation.

### 2.8. Lf Shell-Oily Core Nanocapsules

**Figure 9:**
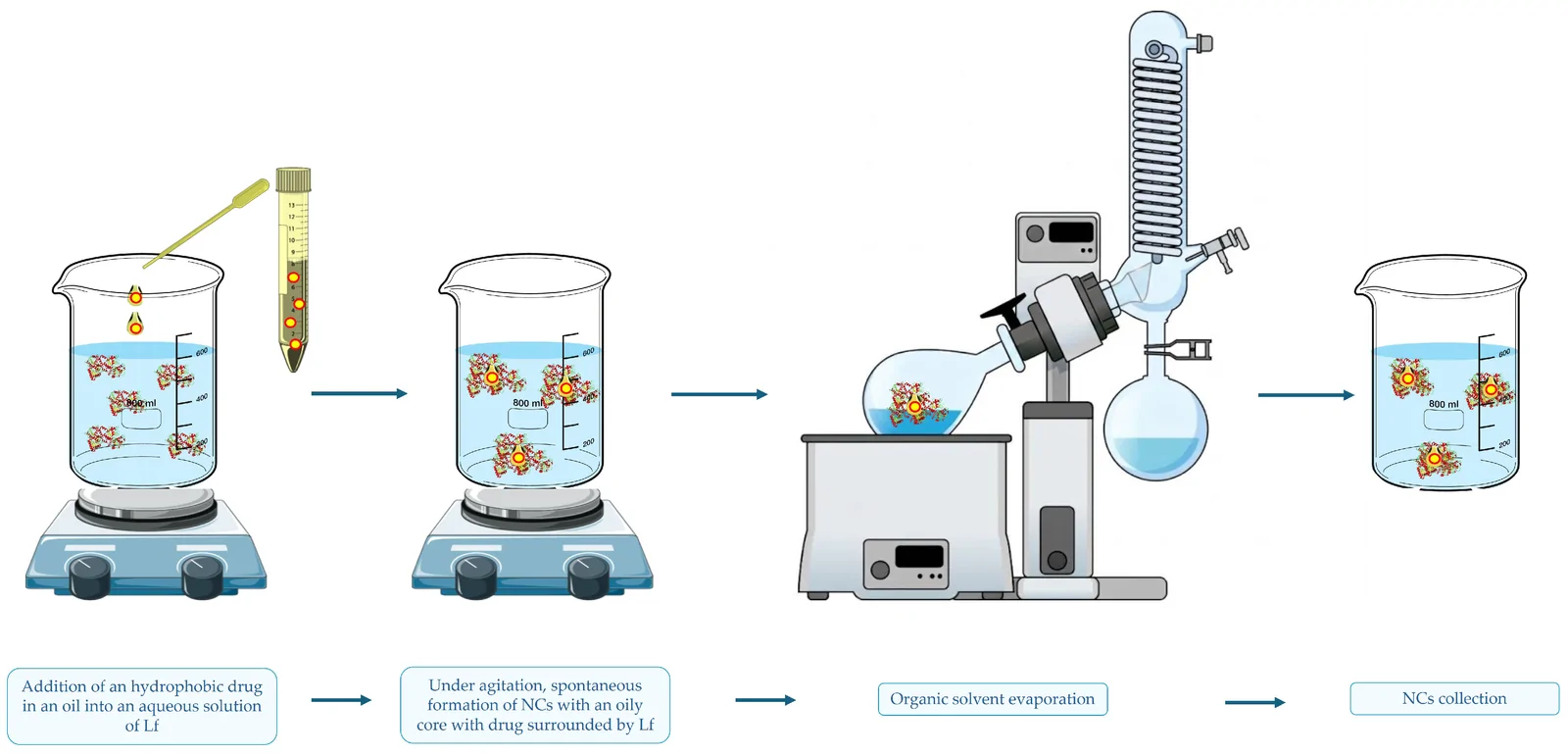
Preparation of lactoferrin shell-oily core nanocapsules.

Polymeric oily-core nanocapsules (NCs) consist of a polymeric protein shell with an oily core for lipophilic drug encapsulation. These systems, which protect active drugs from environmental degradation factors, are widely used for the solubilization and controlled release of hydrophobic compounds. In particular, Lf shell-oily core NCs are biocompatible drug delivery nano-systems (diameter approximately 100–200 nm) for the targeted release of tumor drugs (Table 9) (Figure 9). As a matter of fact, these systems exploit the ability of Lf to bind to receptors overexpressed in tumor tissue, allowing for greater drug absorption. Interestingly, it has also been observed that drugs encapsulated in these structures are more effective, exhibit a longer circulation time, and show reduced toxicity compared to free drugs.

In a study by Abdelmoneem and colleagues, quercetin (QRC), a natural anti-inflammatory and antitumor flavonoid, was co-administered with the targeted therapeutic cancer drug sorafenib (SFB) [63]. NCs were constructed from an oily core composed of capryol-PGMC (propylene glycol monocaprylate) in which the hydrophobic drugs QRC and SFB were solubilized. A cationic Lf shell was then attached to the negatively charged core to form NCs. Additionally, for dual tumor targeting, lactobionic acid (LA) or glycyrrhetinic acid was attached to the Lf shell to interact with asialoglycoprotein (ASGP) and glycyrrhetinic acid receptors on liver cancer cells, respectively. LA/Lf-NCs exhibited a size of 169.0 ± 1.5 nm, a PDI of 0.218, a zeta potential of +8.81 ± 0.51 mV and an EE of 85.6 ± 0.84% and 86.5 ± 0.56% for SFB and QRC, respectively. Glycyrrhetinic acid/Lf-NCs exhibited a size of 230.2 ± 1.7 nm, a PDI of 0.220, a zeta potential of +14.4 ± 0.22 mV and an EE of 88.0 ± 0.32% and 88.0 ± 0.22% for SFB and QRC, respectively. Results from in vitro studies demonstrated increased internalization of dual-targeted LA/Lf NCs in HepG2 (G2 hepatocellular carcinoma) cells compared to Lf and glycyrrhetinic acid/Lf NCs. This dual-targeting strategy enhances delivery specificity to hepatocellular carcinoma, since it can avoid non-specific binding of single-targeted NCs to healthy tissues expressing basal levels of LfR. In contrast, dual-functionalized NCs require the simultaneous engagement of both upregulated Lf receptors and hepatocarcinoma-specific receptors, such as LA or glycyrrhetinic acid receptors. This cooperation among components increases cellular binding affinity and internalization, effectively avoiding off-target binding in healthy tissues. The results of in vivo studies demonstrated that dual-targeted Lf NCs, through the interaction of Lf with its receptors upregulated by tumor cells, had potent antitumor efficacy and reduced hepatotoxicity in mice with diethylnitrosamine-induced HCC [63].

Lipid NCs (LNCs) were also used to encapsulate apocynin (APO), a plant antioxidant with neuroprotective properties, to enhance its effects [64]. Again, since APO has poor bioavailability and is rapidly cleared, to overcome these drawbacks and improve both BBB permeability and brain retention, APO-loaded LNCs were coated with Lf. In this study, lavender essential oil (LAV) was also added to LNCs to enhance their anticonvulsant effects. The resulting LNCs (diameter 59.7 ± 4.5 nm; EE 92 ± 2.4%; and zeta potential of +6.91 ± 2.5 mV) were administered subcutaneously (s.c.) to male Wistar rats. To induce seizures, all animals except the control group received an i.p. injection of pentylenetetrazole (PTZ) 1 h after drug administration. Results of in vivo studies demonstrated that the Lf coating increased bioavailability and brain retention, and that LAV incorporation enhanced antiepileptic activity. Overall, this study evidenced improved anticonvulsant and neuroprotective effects of Lf-APO-LAV/LNCs compared to APO.

### 2.9. Lactoferrin-Inorganic NPs

**Figure 10:**
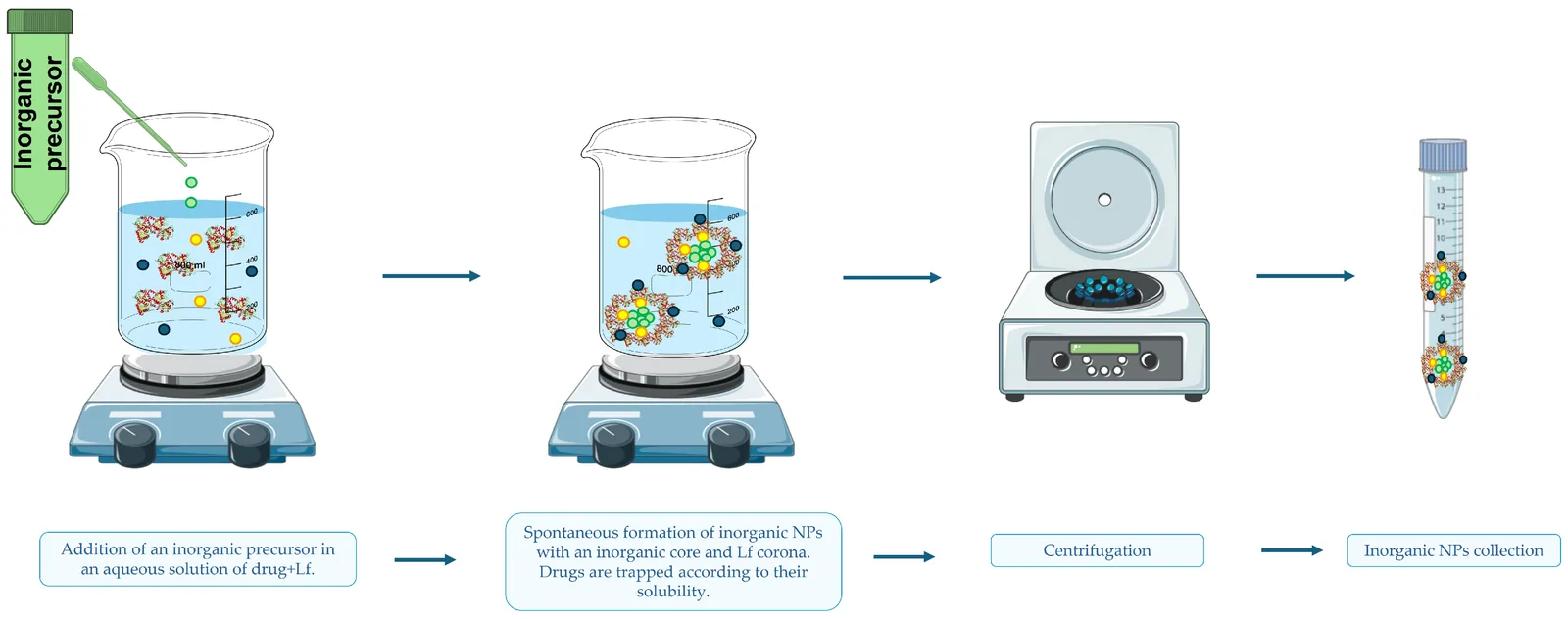
Preparation of lactoferrin-inorganic nanoparticles.

The conjugation of Lf to inorganic NPs is a crucial application leading to hybrid nanoconjugates possessing distinctive properties, including increased loading capacity, ease of ligand binding, and biocompatibility (Figure 10). These hybrid NPs are used in medical imaging and for the targeted delivery of antimicrobial and anticancer drugs (Table 10). Specifically, for cancer therapy, inorganic hybrid NPs can be combined with chemotherapy to achieve a synergistic antitumor effect. Indeed, porous magnetic NPs can absorb magnetic and optical wavelengths, generating heat and killing tumor cells. 

As previously mentioned, much research has focused on the use of NPs as a new therapeutic strategy for brain tumors, where the BBB represents a major obstacle that impedes the passage of most drugs. In particular, the application of nanotechnology in the treatment of gliomas allows for overcoming barriers such as transport across the BBB and the controlled intracellular release of the drug into tumor cells. In this view, Song and collaborators conducted a study in which the surface of graphene oxide/Fe_3_O_4_ NPs (GO@Fe_3_O_4_ NPs) was chemically modified with Lf to enhance their accumulation in glioma cells [65]. After the addition of Lf, in order to utilize its binding capabilities to receptors overexpressed on the surface of tumor cells, the NPs were loaded with doxorubicin hydrochloride (DOX). It was observed that Lf@GO@Fe_3_O_4_@DOX showed higher intracellular release efficiency (acidity-sensitive) and increased toxicity against C6 glioma cells (a rat glial cell line) compared to free DOX or DOX@GO@Fe_3_O_4_. Compared to purely protein-based carriers, inorganic hybrid Lf NPs offer major drug delivery advantages for treating deep-seated tumors, like gliomas. In particular, the combination of Lf with inorganic components enhances the pH-responsive uptake mechanism, minimizing premature drug release outside cells, and helps in pulling the nanocarriers across biological barriers to accumulate anticancer drugs at the tumor site. These results demonstrated the potential use of these Lf nanocomposites for the treatment of gliomas. 

Lf coupled to inorganic NPs is also a promising experimental approach for the targeted treatment of breast cancer. In a study conducted by AbdElhamid and collaborators, theranostic nanocapsules (NCs) based on quantum dots (QDs) were loaded with celecoxib (CXB; EE 94.4%), a specific COX-2 inhibitor with antitumor efficacy, and honokiol (HNK), a natural biphenyl polyphenol compound active against some types of breast cancer [66]. NCs were then chemically coupled to ChS and Lf (Lf–QDs–ChS–NCs, 201.7 nm in diameter, PDI 0.277 and zeta potential +6.16 ± 0.62 mV) via a tumor-cleavable bond to prevent systemic toxicity and enhance tumor accumulation. In vitro studies were carried out on the human breast cancer cell line MCF-7 (Michigan Cancer Foundation-7) in which, through QD-induced fluorescence, NC biodistribution and internalization into tumor cells were followed. The LF–QDs–CS–NCs demonstrated high cellular uptake in MCF-7 cells; furthermore, the dual-drug-loaded nanocomposites exhibited potent anti-angiogenic and apoptotic effects compared to the free drugs. In vivo studies were performed on the EAT (Ehrlich Ascites Tumor) model. These studies were performed by utilizing female BALB/c mice in which the formation of a localized solid mass was induced by subcutaneous injection of EAT cells. The tumor-bearing mice were i.p. injected with the free drugs or the dual-drug-loaded LF–QDs–ChS–NCs. Results of in vivo studies demonstrated that, in addition to showing a significantly higher necrosis score compared to the control group, the LF–QDs–CS–NCs effectively inhibited tumor cell proliferation, successfully localized within tumor tissues, and had a favorable safety profile regarding the kidneys and liver. This multifunctional nanoplatform could serve as a theranostic tool, with QDs acting as probes for fluorescence imaging of tumor cells, thereby facilitating both diagnosis and the monitoring of therapeutic progress.

GBM, a grade IV tumor, is the most malignant and aggressive type of glioma in adults. In the search for new strategies to improve the treatment of GBM, ferroptosis therapy (FT), a therapeutic approach that triggers a type of cell death dependent on iron overload and lipid peroxidation, was developed. In the study conducted by Shen and collaborators, hybrid magnetic NPs (6.6 nm in diameter) of Fe_3_O_4_/Gd_2_O_3_ loaded with cisplatin (CDDP) and conjugated with Lf and RGD dimer (RGD_2_) (FeGd-HN@Pt@Lf/RGD_2_, hydrodynamic size 14.7 nm) were used for FT of orthotopic brain tumors [67]. In this research, human U-87 MG (Uppsala 87 Malignant Glioma) cells were implanted orthotopically into the brain of immunodeficient mice, and FeGd-HN@Pt@Lf/RGD_2_ NPs were i.v. inoculated. Results from in vitro (HBEC-5i cells, a human brain microvascular endothelial cell line) and in vivo studies demonstrated that FeGd-HN@Pt@Lf/RGD_2_ NPs were able to cross the BBB thanks to both their extremely small size and Lf receptor-mediated transcytosis. Once internalized into tumor cells, the NPs release Fe^2+^, Fe^3+,^ and CDDP, accelerating the Fenton reaction and generating reactive oxygen species, thereby inducing tumor cell death. This study demonstrated that FeGd-HN@Pt@Lf/RGD_2_ NPs were able to prevent tumor growth and extended the survival time of treated mice. 

Still seeking treatment strategies for GBM, in another study, Shen et al. developed NPs capable of crossing the BBB for high-contrast magnetic resonance imaging (MRI) and radiosensitization of GBM [68]. In this study, extremely small gadolinium oxide (Gd_2_O_3_) NPs (ES-GON NPs) coated with polyacrylic acid and reduced albumin were synthesized (ES-GON-rBSA; 7.8 nm diameter, zeta potential −22.5 mV) and, subsequently, nanocomposites were obtained by chemically coupling Lf and RGD dimer on the surface of the NPs (ES-GON-rBSA-Lf-RGD_2_, 13.4 nm diameter, zeta potential −15.2 mV). In vitro studies conducted on U-87 MG cells demonstrated that this technology led to improved internalization into tumor cells. Moreover, by this technique, in vitro and in vivo BBB permeation was improved. In vivo studies in subcutaneous (nude mice bearing subcutaneous U-87 MG tumors) and orthotopic GBM models (nude mice bearing orthotopic U-87-Luc or U251-Luc human GBM cell lines expressing the luciferase enzyme) demonstrated that i.v. inoculated ES-GON-rBSA-Lf-RGD_2_ NPs are biocompatible and can accumulate in orthotopic GBM and potentiate GBM radiation therapy, acting as an effective radiosensitizing agent [68].

As mentioned at the beginning of this section, porous magnetic NPs, due to their characteristics, can absorb magnetic and optical energy, generating heat and killing tumor cells. Sharifi and colleagues [69] conducted a study in which maghemite NPs (MMNPs) were synthesized by the hydrothermal method. The NPs were then modified with Lf for tumor targeting and loaded with DOX [69]. 

In this study, the anti-tumor and anti-metastatic activity of DOX-loaded Lf-coated MMNPs in breast cancer was investigated [69]. The results showed that Lf-DOX-MMNPs enhanced DOX antitumor effects in vitro. The drug release profile, measured at pH 6, 6.5, and 7, showed a “burst” pattern in the initial phase followed by slower release, and was significantly faster at pH 6 compared to the other pH levels. Regarding in vivo studies, the breast cancer model was obtained by injecting breast tumor cells into BALB/c mice at the tip of the mammary gland. Coating the surface of the MMNPs with Lf prolonged their blood circulation time. A greater inhibition of tumor growth and a significant increase in targeted drug delivery (TDD) were also observed in mice i.v. injected with Lf-DOX-MMNPs compared to formulations without Lf and DOX alone. Specifically, combined chemotherapy/magnetic field/photothermal (chemo/MF/PTT) therapy inhibited tumor cell proliferation and reduced breast cancer metastasis. Moreover, it has been demonstrated that Lf-DOX-MMNPs were able to accumulate in breast cancer and metastatic lung cancer, preventing significant cytotoxicity in cardiac tissue. This study demonstrated that combined chemo/MF/PTT, together with a pH-sensitive drug delivery system, led to improved antitumor efficacy.

More recent studies on the use of inorganic Lf NPs in the treatment of glioma were conducted by Zhang and colleagues, who loaded a highly cytotoxic Au complex (C2) into Lf NPs (Lf-C2 NPs, mean diameter 58.66 nm, zeta potential −13.6 mV) [70]. Lf-C2 NPs were stable at pH 7.4, whereas at pH 4.7 they exhibited a half-life of 8 h, with up to 76% C2 release after 32 h, demonstrating that release can be regulated via an endosomal/lysosomal mechanism. Results from in vitro studies show that Lf-C2 NPs exhibit high cytotoxicity against U-87-MG glioma cells and induce tumor cell death through apoptosis and autophagy. In vivo studies in a murine system (nude mice bearing an orthotopic U-87 brain tumor) demonstrated that i.v. inoculated Lf-C2 NPs have a remarkable ability to cross the BBB, selectively accumulating in tumor tissue, inhibiting glioma growth, and significantly prolonging the survival rate of tumor-bearing mice without inducing systemic toxicity.

Lf-inorganic NPs have also been used to combat microbial infections, particularly those of bacterial origin. Indeed, it has been observed that hybrid Lf NPs can exhibit different types of antimicrobial activity, offering new approaches in the fight against drug-resistant bacteria (Table 11).

Pomastowski et al. carried out studies on the antibacterial activity of nanocomplexes composed of silver (Ag) and Lf (Lf-Ag NPs) against *P. aeruginosa, S. aureus*, MRSA, and *E. faecalis* [71]. During the formation of nanocomplexes, Ag binding to Lf occurs in two ways: (i) adsorption on the external surface of Lf (Ag-Lf NPs); (ii) internal diffusion and binding within the structure of the Lf globule (Lf-Ag NPs). During internal diffusion, Ag ions bind to the active groups of Lf, forming silver proteins. Aspartic acid and glutamic acid are the amino acids responsible for binding Ag cations via adsorption and subsequent reduction. Concerning the antibacterial activity of Lf-Ag nanocomplexes, results of this study showed that the Lf-Ag NPs were effective against all bacterial strains studied. In particular, *P. aeruginosa*, *S. aureus*, and MRSA were sensitive to the lowest concentration used. Overall, the results demonstrated that the Lf-Ag nanocomplexes can be classified as novel bactericidal agents that can find application in the food and medical industries [71].

It is known that proteins and NPs can interact to create a so-called interfacial protein corona, which confers specific biological activities on NPs. Taking advantage of these properties, further studies on the activity of Lf-Ag NPs were conducted. Nayak et al. produced Lf-Ag NPs and demonstrated that van der Waals interactions and hydrogen bonding allowed Lf to adsorb onto the surface of Ag NPs without altering their stability and shape [72]. The mean hydrodynamic size of the Lf-Ag NPs was 25.44 ± 2.5 nm (for 1 µM Lf) and 32.63 ± 1.72 nm (for 10 µM Lf). Under transmission electron microscopy, the Lf-Ag NPs appeared spherical, with dimensions of 20 ± 10 nm. Moreover, Lf retained its bacteriostatic properties, and the presence of the Lf corona attenuated the cytotoxicity of Ag NPs, while increasing their internalization in the THP-1 human monocytic cell line. Finally, in vivo studies (BALB/c mice) using the protective antigen protein of *Bacillus anthracis* demonstrated that Lf-Ag NPs possess better adjuvant activity than formulations of Ag NPs or Lf alone.

In a study by Abdalla et al., Ag NPs were produced using chitosan and mushroom waste as stabilizing and reducing agents, respectively [73]. The Ag NPs were then surface-functionalized with Lf or graphene oxide (GO) to obtain hybrid NPs with dual antibacterial and antibiofilm activity. The particle sizes of Ag NPs, chitosan-stabilized Ag NPs, GO- Ag NPs, and Lf-Ag NPs ranged from 4 to 15 nm, 7 to 14 nm, 9 to 24 nm, and 10 to 35 nm, respectively. In the absence of chitosan capping, Ag NPs, GO-Ag NPs and Lf-Ag NPs had a PDI of 0.35 ± 0.05, 0.32 ± 0.01, and 0.46 ± 0.11, and a zeta potential of −11.23 ± 0.74 mV, −9.96 ± 1.53 mV, and −11.56 ± 1.50 mV, respectively. In the presence of chitosan capping, Ag NPs, GO-Ag NPs and Lf-Ag NPs had a PDI of 0.29± 0.04, 0.24 ± 0.01, and 0.50 ± 0.01, and a zeta potential of +60.83 ± 1.25 mV, +55.13 ± 0.86 mV, and +52.76 ± 1.26 mV, respectively. Results of this study showed that Lf-Ag NPs and GO-Ag NPs possess similar inhibitory activity against both Gram-positive (*S. aureus*, *Bacillus* spp.) and Gram-negative (*P. aeruginosa*, *E. coli*) bacteria and synergistic efficacy against *P. aeruginosa*, together with synergistic anti-biofilm efficacy against both *S. aureus* and *P. aeruginosa*. Moreover, the use of chitosan as a coating or stabilizing agent enhanced the antibacterial activity of the nanocomposites and cytotoxicity studies on HDF (Human Dermal Fibroblasts) cells demonstrated that Ag-Lf NPs were non-toxic, thus posing an extremely low risk of causing adverse effects.

Later studies, still related to the fight against infectious diseases, focused on the production of Lf NPs as iron transporters. In a study by Alhadide et al., iron oxide Fe_2_O_3_ NPs (IO NPs) and Lf NPs (0.2% *w*/*v*) were synthesized by thermal gelation. The sizes of the IO NPs, Lf NPs, and IO NPs supported on Lf NPs were 80 nm, 60 nm, and 200 nm, respectively [74]. The aim of this study was to develop Lf NPs as iron carriers and to analyze their antimicrobial activity, in combination with whey protein films, against *S. aureus*, *S. agalactiae*, *E. coli*, and *Salmonella enterica*. The results of this study demonstrated that iron oxide NPs were able to inhibit bacterial growth, although the inhibition was lower compared to Lf NPs and Lf NPs loaded on IO NPs. The Lf NPs loaded on IO NPs have also been incorporated into whey protein films, which trap them and act as a biopolymer matrix, leading to the formation of an environmentally friendly packaging material with strong antimicrobial properties. The results of this research also demonstrated that Lf NPs in whey films can be successfully used as antibacterial agents [74].

Finally, concerning viral infections, studies were conducted on the antiviral activity of Lf-coated zinc (Zn) NPs (Lf-Zn-NPs). The Zn particle sizes were around 55.91 nm, and their zeta potentials were −21.62 mV, whereas the Lf-Zn NPs size was 230.8 nm and the zeta potential was −36.85 m. Specifically, Lf-Zn NPs effect on SARS-CoV-2 entry and replication into host cells was investigated in the African green monkey Vero E6 cells [75]. The results of this research demonstrated that Lf-Zn NPs inhibit SARS-CoV-2 entry into host cells by acting on ACE2, the SARS-CoV-2 receptor. Furthermore, in vivo studies in male Wistar albino rats, in which pulmonary fibrosis (PF) was induced by intratracheal injection with bleomycin hydrochloride, demonstrated that oral administration of Lf-Zn NPs significantly improves PF due to their anti-inflammatory, antioxidant and ACE2 inhibitory activities.

## 3. Discussion

According to literature, Lf has been demonstrated to be a useful tool for NPs surface modifications or coatings to improve drug delivery. As a matter of fact, the spectrum of targets in drug delivery is increased by Lf’s multifunctional structure and specific interactions with different receptors. Consequently, Lf may be one of the compounds that fits perfectly into NP-based treatments given the development of different drug carriers for neurological, anticancer and antimicrobial uses.

Lf, which has demonstrated considerable potential in NPs research as a surface material for targeted delivery strategies, can be conjugated with various types of NPs for site-specific release of biomolecules. Indeed, surface modification of NPs with Lf improves bioavailability and stability, while also enabling efficient and targeted cellular uptake, which can improve therapeutic outcomes. However, these Lf-based NPs present not only various advantages but also certain drawbacks that must be addressed before they can be applied in humans.

Regarding the specific characteristics of Lf NPs, key features include: (i) maximal release of the active ingredient at acidic pH (around 5.0) [20]; (ii) the ability to administer hydrophobic drugs via various routes (such as oral, nasal, or intravenous); and (iii) greater efficacy and lower toxicity of the encapsulated drug compared to the drug in its free form. As for the PDI values, they are almost always below 0.3, indicating that the majority of the described NPs represented a homogeneous and monodisperse sample [76]. Concerning the zeta potential of NPs produced via desolvation, electrostatic complexation, and self-assembly methods, as well as inorganic NPs loaded with Lf and pure Lf, the observed values were optimal, indicating the colloidal stability of the NPs in suspension [77,78]. Moreover, Lf NPs prepared using sol-oil methods, thermal denaturation, desolvation, or NAB technology share common advantages, including a size range of 40–200 nm; this allows them not only to avoid renal clearance, thereby extending circulation time and improving the therapeutic index, but also to avoid sequestration in the spleen and liver [7,79,80] (Table S1).

Sol-oil or water-in-oil emulsion method is particularly favored because the preparation of Lf NPs without chemical treatments is considered the method of choice, as it preserves the protein’s native conformation and therefore ensures efficient receptor recognition [8]. Moreover, these NPs offer several advantages, including a small size, high DL and pH-sensitive drug release (which peaks at pH 5.0–6.0). Because the extracellular pH of solid tumors is maintained below 7.0, pH-sensitive NPs are more efficient than conventional counterparts for targeted drug delivery to tumors. In particular, the oil-soil or water-in-oil emulsion method provides stable nano-systems for the delivery of pharmaceutical agents with greater therapeutic efficacy. Specifically, this method allows for the effective solubilization and encapsulation of hydrophobic or poorly water-soluble drugs, forming NPs that protect the active ingredients from enzymatic degradation, oxidation, and hydrolysis. Furthermore, optimized surfactant interactions improve membrane permeability through biological barriers, and the NPs, designed for site-specific accumulation, have reduced systemic toxicity. The advantages of the thermal denaturation method are represented by the fact that this method leads to the formation of highly stable NPs, resistant to both high salt concentrations and rapid pH changes. NPs obtained with this environmentally friendly technique are considered safe carriers for nutrients or drugs [24]. Desolvation process is a widely used method for preparing NPs because the protein carriers are biodegradable, non-antigenic, and minimally toxic. In addition to being cost-effective, this method allows for the good encapsulation of therapeutic compounds with precise control over NPs size, ranging from 100 nm to 200 nm, which is essential for accumulation at target sites [38]. Regarding Nab technique, in addition to high biocompatibility, it reduces allergic reactions and improves drug solubility and pharmacokinetic profile [40]. Regarding electrostatic complexation, it is important to underline that this method is a simple process that requires no complex equipment and, taking place exclusively in an aqueous environment, is highly biocompatible and environmentally friendly. The combination of oppositely charged molecules results in a particularly high drug-carrying capacity, and the ionic bonds holding the NPs together are highly sensitive to changes in pH, ionic strength, or temperature, allowing for targeted drug release [49]. The main advantages of amphiphilic Lf micelles are the increased solubility and bioavailability of the drug and the reduction in side effects, since the controlled release of the drug drastically reduces systemic toxicity. Moreover, NPs synthesis via carbodiimide coupling offers several advantages, such as water solubility and preservation of the protein’s native structure and biological activity [53,54]. Whereas the self-assembling nanoconjugates of Lf and hydrophobic drugs allow the formation of stable protein NPs in aqueous environments, without the need for toxic synthetic polymers. These NPs improve drug solubility, stability, and bioavailability [58]. Finally, the advantages of Lf shell-oily core nanocapsules are due to the presence of an oily core that dissolves and retains high concentrations of hydrophobic drugs, and an Lf shell that increases biocompatibility and slows drug release, ensuring consistent release over time [64]. On the other hand, since it is known that metal NPs can be toxic, the main advantages of conjugating Lf to inorganic NPs are improved biocompatibility, enhanced drug targeting, prolonged drug circulation and, most importantly, reduced toxicity and increased solubility of hydrophobic or poorly soluble inorganic nanomaterials [66] (Table S1).

As previously described, Lf NPs can be successfully used in various fields and have therapeutic applications in both communicable and noncommunicable diseases. In the context of antimicrobial and anti-inflammatory therapy, Lf itself possesses these intrinsic properties, and Lf NPs further enhance efficacy by specifically delivering drugs to infected cells [6]. The antimicrobial efficacy of Lf NPs is due to their ability to penetrate infected cells, delivering higher concentrations of encapsulated drugs and inhibiting pro-inflammatory cytokines [21,45]. 

Concerning cancer treatments, as described above, numerous types of Lf NPs have been developed to overcome the limitations of conventional chemotherapy. For instance, dysregulation of pH homeostasis has been identified as a potential therapeutic weakness [81,82]. In tumor cells, in fact, the intracellular pH is relatively alkaline, favoring their proliferation, while the extracellular space is acidic, favoring malignant progression and contributing to the development of metastases and therapeutic resistance [83]. 

The use of Lf NPs also has significant applications in the treatment of central nervous system disorders, whether of tumoral or non-tumoral origin. Indeed, a key specific advantage of Lf NP-based therapy is the potential for targeted delivery across biological barriers, leveraging the fact that Lf receptors are expressed on various cell types. Specifically, Lf binds to LRP1 located on the plasma membrane of cells in many tissues (liver, brain, lungs, gastrointestinal tract, skin, and connective tissues), enabling Lf-modified NPs to cross protective barriers for targeted disease treatment. For instance, by exploiting these receptors to facilitate drug transport across the BBB, Lf NPs offer novel therapeutic approaches for central nervous system pathologies [84]. Notably, the development of Lf-based micelles capable of crossing the BBB to deliver therapeutic agents for AD has been reported [57].

Lf NPs, however, present some limitations that must be overcome to enable clinical applications. Regarding the synthesis methods, the sol-oil or water-in-oil emulsions require complex production in terms of energy-intensive procedures such as homogenization or ultrasonication. Furthermore, because the high-energy preparation process generates heat, encapsulated drugs may undergo thermal degradation. The high temperatures used for the thermal denaturation method can increase the kinetic energy of the NPs, leading to their agglomeration and deform or destroy the NPs [7]. Moreover, the risk of protein denaturation during preparation and the use of toxic crosslinking agents, which, if not completely removed, can cause adverse reactions during the desolvation process, is a disadvantage [37]. Regarding the Nab technique, disadvantages are related to high production costs, reduced long-term stability, and potential side effects. Instead, the main disadvantage of NPs produced by electrostatic complexation is their poor stability, which can lead to premature drug release. Furthermore, weakly charged or neutral molecules do not bind efficiently, leading to a low DL capacity. A disadvantage of amphiphilic Lf micelles is their minor DL efficiency compared to lipophilic or hydrophobic drug carriers, whereas a further limitation of NPs synthesis via a carbodiimide coupling reaction is its low efficiency and yield [7]. The Lf-hydrophobic drug NPs, while considered promising systems for targeted drug delivery, have significant disadvantages, such as limited colloidal stability and incomplete drug release at the target site. Finally, Lf shell-oily core nanocapsules present some limitations in terms of the structural stability of the shell, which can lead to loss of the oily core. Furthermore, stabilizing the protein shell often requires chemical or physical treatments that could cause toxicity or alter the loaded drug [63]. Conjugating Lf to inorganic NPs can also present some disadvantages; above all, complex purification, which requires laborious and low-yield steps.

Although Lf is an endogenous and therefore inherently biocompatible protein, the synthesis process for Lf NPs can alter their surface conformation or expose structural motifs that the immune system identifies as “non-self” or as material to be eliminated. For this reason, premature clearance mediated by the mononuclear phagocyte system, primarily macrophages residing in the liver and spleen, can represent a major obstacle to the successful systemic administration of Lf NPs. Specifically, the structural changes or partial denaturation of Lf during nanofabrication can trigger opsonization, allowing mononuclear phagocyte system to rapidly capture and clear the NPs from the bloodstream, thereby drastically reducing their therapeutic half-life. Furthermore, the bioavailability of Lf at the target site can be reduced by the coupling of this protein to receptors expressed also on healthy tissues, such as gastrointestinal tract; this results in non-specific binding, leading to increased risk of systemic toxicity. Finally, as with all protein-based nano-formulations, the complexity of the nanocarrier negatively impacts the feasibility of large-scale production and, in some cases, its reproducibility, because the variability of Lf sources can complicate standardization [50]. Indeed, protein source variability, such as the structural and glycosylation differences between commercial bovine Lf and recombinant human Lf, creates unpredictable batch-to-batch variations. However, in the present review, we analyzed only manuscripts describing bovine Lf NPs. The protein purity of commercial bovine Lf is consistently greater than 95%. Furthermore, a sample of industrial lactoferrin may contain LPS at a maximum LPS/lactoferrin molar ratio of 1:1724, meaning that 99.9% of the lactoferrin molecules are free of LPS and possess a glycosylation profile that is stable and constant over time [85,86]. The global standardization of Lf quality is progressing through Novel Food and GRAS in the EU and USA, respectively. It is relatively high grade and may therefore be suitable for research [86]. 

However, without standardized and scalable protocols, minor technical fluctuations during assembly lead to inconsistent NP sizes and unpredictable drug release profiles, which currently prevents their validation in clinical trials. For these reasons, regulatory bodies, such as the FDA and EMA, require advanced physicochemical characterization as well as in-depth evaluations of long-term toxicity and potential immunogenicity [5].

Therefore, although research results are promising, the clinical efficacy of Lf NPs has not yet been demonstrated, and current knowledge is insufficient to support their use. The clinical translation of therapeutic approaches based on Lf NPs is limited by several factors, including nanoparticle synthesis methods, dosage, and routes of administration, making it difficult to compare research findings and hindering standardized application. Thus, although available evidence suggests that Lf NPs hold potential therapeutic value for various pathological conditions, numerous challenges remain regarding formulation optimization, facilitating the transition to pharmaceutical application, and conducting clinical validation. Therefore, at present, it can be stated that Lf NPs represent a promising therapeutic strategy, although they are still under development. Their actual clinical value will depend on further research into optimizing Lf NPs-based drug delivery systems and on validation through clinical trials.

## 4. Conclusions

Lf is a multifunctional protein; Lf NPs can serve as drug carriers for therapeutic applications in both communicable and noncommunicable diseases. Furthermore, Lf’s intrinsic ability to exert antimicrobial and anti-inflammatory activities, independent of the drug it transports, increases its potential for drug-delivery applications. Over the past few decades, numerous studies have been conducted to develop NP-based drug delivery systems, particularly those using Lf NPs, with the aim of overcoming the limitations of conventional therapeutic strategies. Although Lf NPs have been shown to be therapeutically effective, their practical and clinical use has so far been somewhat restricted. When transferring these formulations from the lab to the clinic, researchers encounter a number of biological, translational, and scalability difficulties that are the main cause of this bottleneck. Overall, Lf NPs are expected to increasingly attract the attention of researchers across various fields, such as biology, medicine, and nanotechnology, with the aim of enabling their clinical application.

## Figures and Tables

**Table 1:** Lactoferrin nanoparticles for anticancer drug delivery prepared by sol-oil or water-in-oil emulsion method.

Anticancer Drug Delivery			
Drug	Route of Administration	Major Outcomes	References
DOX	rats with HCC per os, i.v.	Increased bioavailability, minimization of cardiotoxicity, enhanced antitumor effect in rats with hepatocarcinoma	[9,10]
CBDCA	In vitro study neuroblastoma Y79 cell line	Greater antiproliferative activity against Y79 cells compared to soluble CBDCA	[11]
Ce6	In vitro study MDA-MB 231 and SK-OV-3 cell lines	44-fold reduction in the half-maximal inhibitory concentration required to kill cells	[12]
TMZ	Glioma-bearing C57BL/6 mice; i.v.	Better drug pharmacokinetic profile and brain accumulation; reduction in tumor volume and enhanced survival	[13]
AKB (si)RNA	Glioma-bearing C57BL/6 mice; i.v. + free TMZ	Improved antitumor activity and life expectancy in mouse model of glioblastoma. Possible combination with conventional chemotherapy	[8]
5-FU	In vitro study melanoma B16F10 cell line	High encapsulation efficiency, stable and biocompatible delivery vehicle; increased intracellular absorption, and antitumor effect	[14]
5-FU and OX	Wistar rats, i.v.	Reduced systemic toxicity, improved pharmacokinetic profile and antitumor effect in rats with colon adenocarcinoma	[15]
BCNU	In vitro study glioma C6 cell line	Increased cellular uptake and antiproliferative effect	[16]
plasmid pGFPC1 encoding the GFP gene	In vitro study liver cancer HepG2 and glioma GL261 cells lines	Improved gene transmission in cells expressing lactoferrin receptors	[17]

Ce6: Chlorin e6; MDA-MB 231: (M.D. Anderson) Metastatic Breast cell line; SK-OV-3: (Sloan Kettering) human ovarian cancer cell line; TMZ: Temozolomide; 5-FU: 5-Fluorouracil; OX: oxaliplatin; BCNU: carmustine; AKB: Aurora Kinase B; si: small interfering; GFP: Green Fluorescent Protein.

**Table 4:** Lactoferrin nanoparticles prepared by desolvation method.

Drug	Route of Administration	Major Outcomes	References
CUR	In vitro HeLa cells	Identification of optimal conditions for the preparation of protein NPs for drug delivery	[37]
none	In vitro WBC, RBC and PLT	Production of cross-linked NPs that can be used as drug carriers for i.v. and per os administration	[38]
CUR	Sprague-Dawley rats i.n. and i.v.	Improved neuroprotective effects following i.n. administration; brain targeting with i.n. administration superior to that with i.v.	[39]

CUR: curcumin; WBC: white blood cells; RBC: red blood cells; PLT: platelets; i.v.: intravenous; per os: oral; i.n.: intranasal.

**Table 6:** Lactoferrin nanoparticles prepared by electrostatic complexation.

Drug	Route of Administration	Major Outcomes	References
none	-	polysaccharide/Lf NPs for use in the biotechnology, pharmaceutical and food sectors.	[47]
none	-	Temperature stability of Lf/pectin electrostatic nanocomplexes better than Lf alone	[46]
none	-	Formation of Lf NPs for the targeted release of biologically active substances	[48]
CUR	-	Improved water solubility, antioxidant activity and controlled release of hydrophobic CUR	[49]
DOX, EA	In vitro: A549 lung cancer cells and in vivo: lung tumor-bearing mice	Inhalable Lf-ChS NPs as a vector for targeted drug delivery against lung cancer	[50]
none	In vitro *S. aureus* and *E. coli*	G/Lf NPs possess greater antimicrobial activity than Lf; potential applications as a food preservative.	[51]

CUR: curcumin; DOX: doxorubicin; EA: ellagic acid; ChS: chondroitin sulfate; G: gellan gum.

**Table 8:** Lactoferrin-drug nanoconjugates.

Drug	Route of Administration	Major Outcomes	References
DFX	In vitro: PC12 cells; In vivo: AD Wistar rat model i.p.	Neuroprotective effect of deferasirox conjugated to Lf in the management of neurodegenerative diseases through metal chelation therapy	[58]
CUR	In vitro: HCT116 cells	The conjugation of CUR with Lf results in a drug delivery system that acts on tumor cells through active and passive targeting	[59]
DOX	In vitro: DU145 prostate cancer cell cells In vivo: per os TRAMP mice	The potential of a new and safer Lf-DOX conjugate to combat drug resistance is highlighted	[60]
PMT and EA In MSNPs	In vitro MCF-7 breast cancer cells	The synergistic combination demonstrated a promising delivery strategy for breast cancer therapy by overcoming drug resistance and minimizing systemic toxicity	[61]
RIF	In vitro: *E. coli*, MM or MRSA-infected macrophages in vivo: i.v. BALB/c mice	High therapeutic efficacy in disease models such as subcutaneous infections, sepsis, and bacterial keratitis	[62]

DFX: deferasirox; PC12: rat pheochromocytoma cell line; AD: Alzheimer’s disease; i.p.: intraperitoneal; CUR: curcumin; HCT116: human colon tumor cells; DOX: doxorubicin; per os: oral; TRAMP: transgenic mouse prostate adenocarcinoma; DU145: Duke University prostate cancer cells; PMT: pemetrexed; EA: ellagic acid; MSNPs: mesoporous silica nanoparticles; MCF-7: Michigan Cancer Foundation-7 breast cancer cells; RIF: rifampicin; i.v.: intravenous; *E. coli*: *Escherichia coli*; MM: *Mycobacterium marinum*; MRSA: methicillin-resistant *Staphylococcus aureus*.

**Table 9:** Lactoferrin-coated lipid nanocapsules.

Drug	Route of Administration	Major Outcomes	References
QRC and SFB, LA or glycyrrhetinic acid	i.v. DEN-induced HCC mice model	Powerful antitumor efficacy and reduced hepatotoxicity	[63]
APO, LAV	s.c. Wistar rats	Enhanced anticonvulsant and neuroprotective effect of Lf-APO-LAV/LNCs compared to APO	[64]

QRC: quercetin; SFB: sorafenib; LA: lactobionic acid; i.v.: intravenous; DEN: diethylnitrosamine; HCC: hepatocellular carcinoma; APO: apocynin; LAV: lavender essential oil; s.c.: subcutaneous; LNCs: lipid nanocapsules.

**Table 10:** Lactoferrin-inorganic nanoparticle for anticancer drug delivery.

Anticancer Drug Delivery				
Inorganic NPs	Drug	Route of Administration	Major Outcomes	References
GO@Fe_3_O_4_ Nano-composites	DOX	In vitro C6 glioma cells	Higher intracellular release and cytotoxicity of Lf@GO@Fe_3_O_4_@DOX compared to free DOX or DOX@GO@Fe_3_O_4_.	[65]
QDs-NCs	CXB HNK	In vitro: MCF-7 breast cancer cells in vivo: i.p. EAT model	increased cytotoxicity, and marked reduction in tumor volume	[66]
Fe_3_O_4_/Gd_2_O_3_ NPs	CDDP	In vivo: i.v. nude mice bearing orthotopic GBM	Synergistic tumor cell growth inhibition, higher response to FT	[67]
Gd_2_O_3_ NPs	None	In vitro: U-87 MG cells; in vivo: i.v. mice bearing s.c. and orthotopic GBM	Ultra-BBB permeable and biocompatible radiosensitizing agents enhancing radiotherapy effect	[68]
MM NPs	DOX	i.v. mice bearing breast tumors	Lf-DOX-MMNP accumulates in breast cancer and lung metastasis, with greater tumor inhibition compared to formulations without Lf and DOX alone. Combined MF-PTT therapy inhibited tumor growth.	[69]
Gold cytotoxic complex (Au III) NPs (Lf-C2)	None	In vivo: i.v. nude mice bearing orthotopic brain tumor	reduced systemic toxicity and greater glioma growth inhibition compared to free C2	[70]

GO: graphene oxide; DOX: doxorubicin hydrochloride; QDs; Quantum dots; NCs: nanocapsules; CXB: celecoxib; HNK: honokiol; MCF-7: Michigan Cancer Foundation-7; EAT: Ehrlich Ascites Tumor; i.p.: intraperitoneal; Fe_3_O_4_: iron (II,III) oxide; Gd_2_O_3_: gadolinium(III) oxide; CDDP: cisplatin; i.v.: intravenous; GBM: glioblastoma multiforme; FT: ferroptosis; s.c.: subcutaneous; U-87 MG: Uppsala 87 Malignant Glioma; MF: chemo-magnetic field; PTT: photothermal therapy; MM: Mesoporous Maghemite.

**Table 11:** Lactoferrin-inorganic nanoparticle for antimicrobial drug delivery.

Antimicrobial Drug Delivery				
Inorganic NPs	Drug	Route of Administration	Major Outcomes	References
Ag NPs	None	In vitro *P. aeruginosa*, *S. aureus*, *E. fecalis*, and MRSA	New economically and environmentally sustainable antimicrobial agents	[71]
Ag NPs	None	In vitro: THP-1 cells, *E. coli*, *S. aureus* In vivo: mice immunized i.p.	Lf layer decreased Ag NP cytotoxicity and enhanced the humoral immune response	[72]
Ag NPs	None	In vitro: HDF cells, *S. aureus*, *Bacillus* spp., *P. aeruginosa* and *E. coli*	Enhanced antibacterial and antibiofilm activity, together with reduced cytotoxicity.	[73]
IO NPs	None	In vitro *S. aureus*, *S. agalactiae*, *E. coli*, and *Salmonella enterica*	Lf-IO NPs in whey films exhibit effective antibacterial activity	[74]
Lf-Zn NPs	None	In vitro, Vero E6 cells; in vivo: Bleomycin-induced PF Wistar albino rats (i.t.); NPs (per os)	greater safety, better cellular absorption and therapeutic efficacy	[75]

Ag: silver; MRSA: Methicillin-resistant *Staphylococcus aureus*; THP-1: Tohoku Hospital Pediatrics-1; i.p.: intraperitoneally; HDF (Human Dermal Fibroblasts; IO NPs: iron oxide Fe_2_O_3_; Lf-Zn NPs: Lf-coated zinc NPs; Vero E6: *Verda reno* E6; PF: pulmonary fibrosis; i.t.: intratracheal injection; per os: oral.

## Data Availability

No new data were created or analyzed in this study. Data sharing is not applicable to this article.

## Supplementary Materials

The following supporting information can be downloaded at: https://www.mdpi.com/article/10.3390/ijms27209226/s1.
