# Supplementary material for: Lactoferrin and Nanotechnology: New Perspectives and Therapeutic Applications for This Multifunctional Protein
Source: Int J Mol Sci. 2026 Oct 17;27(20):9226. doi: 10.3390/ijms27209226 (PMC13705528; doi:10.3390/ijms27209226)
Supplement: Supplementary file 1 [file ijms-27-09226-s001.zip › ijms-4600285-supplementary.pdf]

Table S1. Comparison of preparation techniques for lactoferrin-based nanocarriers

Preparation techniques	NPs size (nm)	EE%	PDI	ζ mV	DL%	Stability	Cross-linking agent or solvent residues	Scalability/ reproducibility	Toxicity	Route of administration	Release kinetics	Targeting efficiency	Ref.
**Sol-oil or water-in-oil emulsion**	40 – 140	high 58-79	medium 0.17 - 0.33	-2 -24	high 31.2	good	low moderate	good	low	per os, i.v., topic	sustained release	average	[8-17, 19-23]
**Thermal denaturation**	67 – 177	high 50->99	medium 0.21 - 0.47	+22	low 1.4-2.7	high pH-resistant structure	none	very high simple/scalable process	no	per os, i.v., topic	slow and sustained, excellent gastric resistance	reduced/ medium	[24-26, 36]
**Desolvation**	85 – 200	high 54->90	low 0.07 - 0.3	+22 +40	standard 9.6	high (after the cross-linking process)	very high	moderate, rate of addition sensitive	low	per os, i.v,. i.n.	very slow, related to the degree of cross-linking	high	[37-39]
**Nab-technique**	100 - 200	very high >90	low 0.10 - 0.20	+8 +27	standard 12.44	reduced long-term stability	none	high (high-pressure homogenization technology)	no	per os, i.v.	rapid/sustained dependent on non-covalent dissociation of the protein complex	high	[40, 41, 45]
**Electrostatic complexation**	92 – 400	very high >90%	medium 0.18 - 0.30	-40 +21	low < 3%	moderate	none	very high (immediate mixing)	no	i.n.	immediate for pH changes or ionic competition	high	[46-50, 51]
**Amphiphilic micelles**	100 - 230	high 62->90	medium 0.20 - 0.30	-32 +41	moderate 6-9	high (above the CMC)	low moderate	average	no	i.v., per os	diffusion-controlled release (CMC depending)	very high	[53,54, 56,57]
**Hydrophobic moieties Self-assembly**	100 - 500	high often >85	medium 0.16 - 0.47	+9 +15	very high often > 30	excellent	none	low-medium	no	per os, i.v., i.p.	intracellularly activated	incomplete drug release at the target site	[58-61]
**Shell-oily core**	169 - 230	very high often>90	low 0.2- 0.22	+7 +14	high 15-30	low, shell instability can lead to oily core loss	low moderate	high (spontaneous emulsification)	very low	i.v., s.c.	prolonged, slowed by lactoferrin shell	high	[63, 53]
**Lactoferrin-inorganic NPs**	13 - 230	high 50-94	medium 0.27- 0.50	-37 +52	low < 3%	excellent, highly stable inorganic core	low moderate	average	very low	per os, i.v., i.p, i.t.	prolonged, triggered or remote-controlled release	Excellent: receptor targeting and optical/theranostic properties	[65-75]
**Pure lactoferrin**	150 - 250	-	medium 0.20 - 0.30	-52 +33	-	average, subject to enzymatic degradation	none	very high simple and scalable process	no	per os, i.v., i.n.	-	high	[79]

NPs: nanoparticles; E: encapsulation efficiency; PDI: polydispersity index; ζ: zeta potential; DL: drug loading; CMC: critical micelle concentration; per os: oral; i.v.: intravenous ; i.n.: intranasal; i.p. : intraperitoneal ; s.c.: subcutaneous; i.t.: intratracheal injection.
